# Zebrafish *con/disp1 *reveals multiple spatiotemporal requirements for Hedgehog-signaling in craniofacial development

**DOI:** 10.1186/1471-213X-9-59

**Published:** 2009-11-30

**Authors:** Tyler Schwend, Sara C Ahlgren

**Affiliations:** 1 Integrated Graduate Program, Northwestern University Feinberg School of Medicine, 303 E. Chicago Avenue, Chicago, IL, 60611, USA; 2 Department of Pediatrics, Northwestern University Feinberg School of Medicine, 2300 Children's Plaza, Chicago, IL, 60614, USA; 3 Developmental Biology Program, Children's Memorial Research Center, 2400 Children's Plaza, Chicago, IL 60614, USA

## Abstract

**Background:**

The vertebrate head skeleton is derived largely from cranial neural crest cells (CNCC). Genetic studies in zebrafish and mice have established that the Hedgehog (Hh)-signaling pathway plays a critical role in craniofacial development, partly due to the pathway's role in CNCC development. Disruption of the Hh-signaling pathway in humans can lead to the spectral disorder of Holoprosencephaly (HPE), which is often characterized by a variety of craniofacial defects including midline facial clefting and cyclopia [1,2]. Previous work has uncovered a role for Hh-signaling in zebrafish dorsal neurocranium patterning and chondrogenesis, however Hh-signaling mutants have not been described with respect to the ventral pharyngeal arch (PA) skeleton. Lipid-modified Hh-ligands require the transmembrane-spanning receptor Dispatched 1 (Disp1) for proper secretion from Hh-synthesizing cells to the extracellular field where they act on target cells. Here we study *chameleon *mutants, lacking a functional *disp1*(*con/disp1*).

**Results:**

*con/disp1 *mutants display reduced and dysmorphic mandibular and hyoid arch cartilages and lack all ceratobranchial cartilage elements. CNCC specification and migration into the PA primorida occurs normally in *con/disp1 *mutants, however *disp1 *is necessary for post-migratory CNCC patterning and differentiation. We show that *disp1 *is required for post-migratory CNCC to become properly patterned within the first arch, while the gene is dispensable for CNCC condensation and patterning in more posterior arches. Upon residing in well-formed pharyngeal epithelium, neural crest condensations in the posterior PA fail to maintain expression of two transcription factors essential for chondrogenesis, *sox9a *and *dlx2a*, yet continue to robustly express other neural crest markers. Histology reveals that posterior arch residing-CNCC differentiate into fibrous-connective tissue, rather than becoming chondrocytes. Treatments with Cyclopamine, to inhibit Hh-signaling at different developmental stages, show that Hh-signaling is required during gastrulation for normal patterning of CNCC in the first PA, and then during the late pharyngula stage, to promote CNCC chondrogenesis within the posterior arches. Further, loss of *disp1 *disrupted normal expression of *bapx1 *and *gdf5*, markers of jaw joint patterning, thus resulting in jaw joint defects in *con/disp1 *mutant animals.

**Conclusion:**

This study reveals novel requirements for Hh-signaling in the zebrafish PA skeleton and highlights the functional diversity and differential sensitivity of craniofacial tissues to Hh-signaling throughout the face, a finding that may help to explain the spectrum of human facial phenotypes characteristic of HPE.

## Background

The vertebrate head skeleton is largely derived from cranial neural crest cells (CNCC) which originate in the dorsal neural tube and migrate ventrally to populate the pharyngeal arches (PA) [3]. The final skeletal pattern depends on intrinsic positional information acquired by CNCC prior to migration [4-6], instructive signals encountered by CNCC during migration [7-9], and morphogenetic cues that CNCC receive from surrounding tissues upon completing migration [10,11]. There are seven paired skeletal elements that arise on either side of the midline. The CNCC of the first PA (mandibular) forms the jaw, while those in the second PA (hyoid) add support to the jaw structure. Posterior to these two PA, the CNCC populate five posterior PA to form bilateral gill support structures. Although all of these elements derive from CNCC, the neural crest arise from different anterior-posterior regions of the neural axis, travel in different streams to arrive at the final destination, express different Hox genes, and encounter different local environments at the point of condensation [6,12]. However, the genes that regulate the production of cartilage from CNCC are shared regardless of location [13].

Genetic studies have revealed conserved requirements for Hedgehog (Hh)-signaling in craniofacial development. Abrogation of Hh-signaling through inactivation of the Hh-receptor *Smoothened *(*Smo*) leads to a complete loss of cranial skeletal elements in zebrafish and mouse [14,15]. It has been pointed out that these animal models represent extreme phenotypes, with complete suppression of signaling, and often result in embryonic lethality [2]. However, mutations within the key Hh-family member, Sonic Hedgehog (SHH) in humans can lead to the spectral disorder of Holoprosencephaly (HPE), often characterized by a variety of craniofacial defects including midline facial clefting and cyclopia [1]. It has been further suggested that HPE can be found in patients with significant impairment of the Hh-signaling pathway rather than its complete elimination [2].

Genetic and pharmacological vertebrate studies have revealed that Hh-signaling is required by multiple facial tissues, including the neural crest, and the pathway's action is needed at multiple times during embryonic development in order for the face to grow and pattern normally [16-23]. Thus, it is becoming apparent that the range of phenotypes that are apparent in HPE may be in part due to the affected tissue(s) and the timing of the Hh-pathway disruption.

The precise role for Hh-signaling in the zebrafish PA skeleton has yet to be elucidated. In the dorsal neurocranium, Hh-signaling has been demonstrated to control the placement and condensation of CNCC upon facial ectoderm prior to their chondrification [18]. This CNCC patterning defect is followed by a delay in the expression of the chondrogenic marker *sox9a*, or a complete absence of gene expression in more severe genetic backgrounds where *smo *is disrupted [18,23]. Neurocranial and upper jaw cartilage elements, including the ethmoid plate, paired trabeculae and pterygoid process of the palatoquadrate, are lost or mislocalized with treatments of Cyclopamine (Cya), a pharmacological antagonist of Hh-signaling, administered to whole embryos during the gastrulation period, prior to CNCC migration [18,23]. This requirement is likely cell non-autonomous as the oral ectoderm requires early Hh-signals in order to promote CNCC condensation and the expression of growth factors as revealed by genetic mosaic analysis [18]. In addition to this, Wada et al., 2005 has proposed a second, cell-autonomous requirement for Hh-signaling wherein the Hh-signal is secreted, likely by the oral ectoderm, and is required by post-migratory CNCC that will form the neurocranium to undergo chondrogenesis. This finding is supported in mice and zebrafish with a tissue-specific loss of *Smo *function in CNCC, as these cells become properly patterned in the face but rarely differentiate into cartilage tissue [18,22]. Despite this, these studies do not explain what happens to the CNCC after they fail to condense.

This study seeks to expand our understanding of Hh-signaling in craniofacial development by presenting a detailed characterization of ventral cartilage development in the PA upon genetic inactivation of the Disp1 gene by using the *chameleon *(*con/disp1*) mutant zebrafish. Disp1 acts within Hh-synthesizing cells to disperse Hh-ligands into the extracellular field where they are received by Hh-target cells [24]. We found that these mutant fish demonstrate a significant, but not complete, reduction in Hh-signaling, similar to what has been proposed for HPE patients with DISP1 mutations [2]. Furthermore, we pinpoint precisely during development when Hh-signals are required for ventral cartilage development through pharmacological inhibition using Cya. Collectively we report findings that provide insight into Hh-signaling function during jaw and gill cartilage formation in the developing zebrafish pharyngeal arch skeleton.

## Methods

### Animals

Zebrafish (*Danio rerio*) embryos were obtained from natural crosses and staged as previously described [25]. Homozygous *con/disp1*^*tm*15*aandb*392 ^were identified among their siblings by PCR genotyping prior to 24 hours post fertilization (hpf) (detailed genotyping strategy for *con/disp1*^*tm*15*a *^was reported in Nakano et al., 2004, and the genotyping strategy for *con/disp1*^*b*392 ^is reported in this manuscript) or by somite morphology at later stages [26]. Genotyping primers: *con*^*tm*15*a*^_Forward, 5'CCAGCTGTGGTGGTCTTACA3', *con*^*tm*15*a*^_Reverse, 5'CAGAGGCTTCTTCGCAGTCT3'; *con*^*b*392^_Forward, 5'CCAAAGATCATTACGATCGAAAGAATCGA3', *con*^*b*392^_Reverse, 5'CCATGTGGTCTCCCATAGTCATTA3.' Next, restriction enzyme reactions were performed to differentiate PCR products, for *contm15a *we used *PvuII *(New England Biolabs) and for *b392 *we used *ClaI *(New England Biolabs) which both digest mutant alleles (see additional file 1 for further explanation). *fli1*:gfp transgenic zebrafish expressing the gfp-transgene under the control of the Fli1 promoter was previously characterized [27]. Wild type refers to *con/disp1*^+/+,+/- ^siblings, except in Cya-treatment experiments wherein the Tübingen strain was utilized.

### Alcian blue staining

Cartilage analysis was performed as previously described, zebrafish larvae were fixed at 96 hpf and treated with Alcian blue solution dissolved in 80% ethanol/20%glacial acetic acid (acid alcohol) for several hours or overnight. Larvae were destained in several washes of acid alcohol before being transferred to a 1% KOH:3% hydrogen peroxide solution for further clearing of pigment cells. Larval tissue was then digested in trypsin, followed by dissection of cartilages which were then flat mounted as previously described [28]. Cartilage preparations were visualized on a Leica MZ16F microscope with camera.

### Whole mount in situ hybridization, immunohistochemistry and histology

Whole-mount *in situ *hybridization was conducted essentially as previously described [29]. Embryos fixed at a stage older than 25 hpf were raised in 2 mM 1-phenyl-2-thiourea in embryo medium to prevent pigmentation. Embryos were visualized on a Leica MZ16F microscope with camera. Images were processed using Adobe Photoshop CS2.

Immunohistochemistry was performed as previously described [30]. The Zn5 antibody (Zebrafish International Resource Center [ZIRC], Oregon) was used at a 1:500 dilution in goat serum blocking solution (ZIRC, Oregon). Confocal stacks of the embryos were generated on a Zeiss LSM510.

For histological preperations, gelatin-embedded embryos were sectioned at 10 μm on a Leica cryostat and subjected to Gill's 2× Hematoxylin and Eosin (Fischer Scientific). Embryos were visualized on a Leica DMR-HC upright microscope.

### TUNEL staining

For cell death studies, embryos fixed for analysis at a stage older than 18 hpf were incubated in 1 μg/mL Proteinase K solution to solubilize tissues and washed twice in PBS-Tween (PBST). Embryos were then incubated in the labeling and enzymatic solutions that are provided by the manufacturer of the In Situ Cell Death Detection Kit (Roche) for 1 hour at 37°Celcius. Enzyme and label solution consists of TdT (terminal deoxynucleotide transferase-mediated dUTP) and fluorescein-conjugated deoxynucleotides in buffer. After three washes in PBST to remove non-specific labeling, embryos were bathed in blocking solution (2% BSA, 1% DMSO in PBST) for 1 hour at room temperature, followed by incubation in blocking solution containing an anti-fluorescein-conjugated alkaline phosphatase (Roche) overnight at 4°Celcius. The following day, embryos were washed multiple times with PBST and incubated at room temperature in an NBT/BCIP developing solution (Promega) to visualize dying cells. Stained embryos were visualized on a Leica MZ16F.

### Cyclopamine treatments

Cya (LC Laboratories) was solubilized in 95% ethanol to obtain a stock solution of 50 mM, which was further diluted in zebrafish embryo water to a working stock concentration of 100 μM. Embryos were bathed in the Cya working stock for 4-24 hours at a time. Immediately following treatment, the Cya solution was removed, embryos were washed in multiple rounds of zebrafish embryo water, and then allowed to develop further until 48-96 hpf, wherein they were analyzed by *in situ *hybridization or cartilage staining.

### qPCR analysis

Total RNA extraction was performed using RNA Tissue Extraction and DNase kits (Versagene). cDNA was synthesized using SuperScript III and random primers (Invitrogen). PCR reactions were performed using Platinum SYBR Green qPCR Super Mix-UDG (Invitrogen) on a MJ Research Chroma-4 PCR machine and primers: gli1_Forward: 5'CAGACGTCCTCTCGCCTTAC3,'

gli1_Reverse: 5'AGTAGCGCTGTCCTTGCATT3,' disp1_Forward: 5'GCTGTAGGGCTTTCTGTGGA3,'

disp1_Reverse: 5'GCCACTGTTGGTAGGAGCAT3,' gapdh_Forward: 5'GAAGGTGGGAAACTGGTCAT3,' gapdh_Reverse: 5'TTGCACCACCCTTAATGTGA3.' Genes being analyzed were then normalized to *gapdh *levels and relative quantification of gene expression was calculated using the Pfaffl method variation on 2^-ΔΔCt ^[31], displaying data as fold difference in mutants relative to wild type.

### RNA Microinjections

Plasmids to generate full-length *shh *and Flag-tagged *shh*N (N terminus of *shh *fused to C-terminal Flag epitope), which encodes unmodified *shh*, have been previously described [26,32]. To synthesize capped mRNAs, the plasmids were linearized downstream of the insert, followed by transcription reactions using the Ambion mMESSAGE mMACHINE kit according to manufacturer's instructions. ~1 nanoliter (nl) of 100-150 pg/nl mRNA, suspended in 0.2 M KCl, was pressure-injected at 1-2 cell stage. Embryos were then allowed to develop to 96 hpf, wherein they were analyzed by cartilage staining.

## Results

### Requirement for Hh-signaling in ventral cartilage development

Zebrafish lacking the Hh-signaling pathway, due to *smo *loss, display complete absence of PA-derived cartilages [14]. To better understand the role of Hh-signaling in this process, we studied cartilage development within two *con/disp1 *mutant alleles: the *con/disp1*^*tm*15*a *^(genetic mutation characterized in [26]) and *con/disp1*^*b*392 ^(genetic mutation described in this manuscript, see additional file 1). *con/disp1 *mutant phenotypes are less severe than *slow-muscle-omitted *(*smu/smo*) mutant phenotypes, making them more amenable for detection of subtle deficits [33]. *con/disp1*^*tm*15*a *^and *con/disp1*^*b*392 ^alleles displayed similar penetrance in their craniofacial phenotype, and also shared similar gene expression reductions in *gli1*, a Hh-signaling pathway transcription factor that is sensitive to the pathway's attenuation (additional file 1). Due to the similar nature of the mutant phenotypes, and in an effort to be concise, in this study we simply report our findings using the *con/disp1*^*tm*15*a *^allele, which we will herein refer to as *con/disp1*.

**Figure 1:**
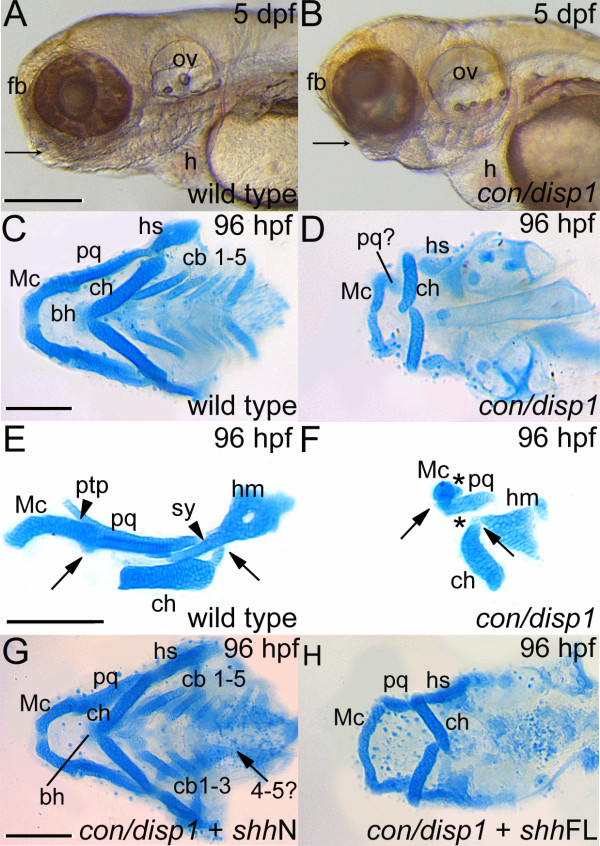
***con/disp1 *mutants display defective cartilage development in the PA which is partly rescued by RNA encoding non lipid-modified Shh**. (**A,B**) Lateral views of live larvae show a reduction in head tissue and a deficit in jaw outgrowth (arrowhead) in *con/disp1 *mutant larvae at 5 dpf. (**C**) Ventral view of 96 hpf Alcian blue-stained wild type cartilages. (**D**) *con/disp1 *mutant cartilages reveal reduced, hypoplastic mandibular and hyoid arch cartilage elements and a complete absence of the cb 1-5 elements and midline-forming bh cartilage. (**E,F**) Lateral view of jaw cartilages reveals malformed joints (arrows denote joint site), fewer chondrocytes contributing to the pq cartilage element and a complete loss of the ptp and sy cartilage element in *con/disp1 *mutant (arrowhead denotes presence of cartilage, asterisk denotes cartilage absence). (**G**) Injection of 150 pg *shh*N mRNA into genotype-confirmed *con/disp1 *mutant mostly restores cartilage elements to wild type state (arrow indicates unilateral rescue in cb 4-5 elements), while (**H**) *con/disp1 *mutant injected with 150 pg *shh*FL mRNA are phenotypically similar to noninjected *con/disp1 *larvae. Scale bar: 50 μM.

5 day old *con/disp1 *mutants were distinguished by an overall reduction in head size and displayed micro- or agnathia due to reduced jaw outgrowth (arrow, Figure 1A, B) and significant heart edema when compared to wild type larvae (Figure 1A, B). A closer examination of cartilage formation at 96 hpf using Alcian blue staining revealed the mandibular arch cartilages (lower jaw) in wild type larvae to be completely patterned, including the ventral Meckel's cartilage (Mc) and dorsal palatoquadrate (pq) (Figure 1C). However, *con/disp1 *Mc and pq elements were significantly reduced (Figure 1D). Hyoid arch cartilages, which support the lower jaw, contain the dorsal hyosymplectics (hs) and ventral ceratohyals (ch). *con/disp1 *hs and ch cartilages were smaller and dysmorphic, with the ch often inverted posteriorly (Figure 1C, D). Inversion of midline arch elements is a common developmental byproduct of severe posterior arch cartilage disruptions [34] and strikingly the ceratobranchial (cb) cartilages of the five posterior arches (gill-support) were consistently absent in the *con/disp1 *larvae (Figure 1C, D). Closer examination of the remaining *con/disp1 *jaw elements revealed a complete loss of dorsal cartilage elements comprising the pterygoid process (ptp) of the mandibular arch and the symplectic (sy) of the hyoid arch (arrowhead marks cartilage elements in wild type embryos, asterisk denotes cartilage loss in mutants, Figure 1E, F). Additionally, lateral views of cartilage preparations revealed malformed joints in both anterior arches (arrows in Figure 1E, F)

Disp1 regulates the release of lipid-modified Hh-molecules from their site of synthesis [26]. To determine if Disp1 action is conserved in the craniofacial skeleton, we compared the outcome on *con/disp1 *cartilages upon ectopic mRNA expression of lipid-modified Shh (*shh*FL) or the N-terminal domain of Shh (*shh*N), which retains Hh-signaling capacity but fails to become lipid-modified. Injecting offspring from an incross mating of *con/disp1 *heterozygotes with 150 pg *shh*FL mRNA at the 1-cell stage yielded no discernable differences in cartilage phenotypes (11/45 [24%] larvae showing *con/disp1*^-/- ^cartilage defects) from their uninjected siblings (15/55 [26%] larvae) (Figure 1H), likely due to the inability of lipid-modified-ShhFL protein to be secreted from *disp1*^-/-^mutant cells. In contrast, injecting *con/disp1 *heterozygote offspring with 150 pg of *shh*N mRNA reproducibly decreased the number of larvae displaying PA-derived cartilage defects (3/50 [6%]) compared to uninjected siblings (12/45 [25%]). We determined cartilage rescue in these studies to be a partial-to-full restoration of cartilage outgrowth in the mandibular arch coupled with partial restoration of cb cartilages (arrow in Figure 1G shows unilateral rescue in cb 4-5) in genotype-confirmed *con/disp1 *mutants (Figure 1G). Failure of *shh*N to fully restore *con/disp1 *cartilage defects could be attributed to a loss of potency by non-lipid-modified versions of Shh to activate Hh-target genes [35]. Alternatively, because *shh*N completely restored cartilages in the anterior arches, loss of full cartilage restoration may be due to the observation that arch cartilages develop sequentially, with posterior elements forming last, thus allowing for ectopic mRNA dilution and degradation to occur prior to the time in which chondrogenesis has completed [36].

### Tracking *con/disp1 *CNCC development with the *fli1:gfp *transgene

PA cartilages are derived from CNCC in zebrafish, consistent with higher vertebrates [37,38]. Thus, cartilage defects could be due to either a failure of migrating CNCC to populate arch primordia or to a lack of postmigratory-CNCC differentiation or survival. Others have reported that zebrafish with Hh-signaling disruptions display no discernable disruptions to CNCC specification or migration into the arch primordia [18,23]. Like these reports, we found that *con/disp1 *mutants were identical to wild type embryos in CNCC specification (12 hpf) and early migration (14-22 hpf) into the PA as visualized by neural crest markers *foxd3 *and *crestin *(additional file 2) [39,40].

**Figure 2:**
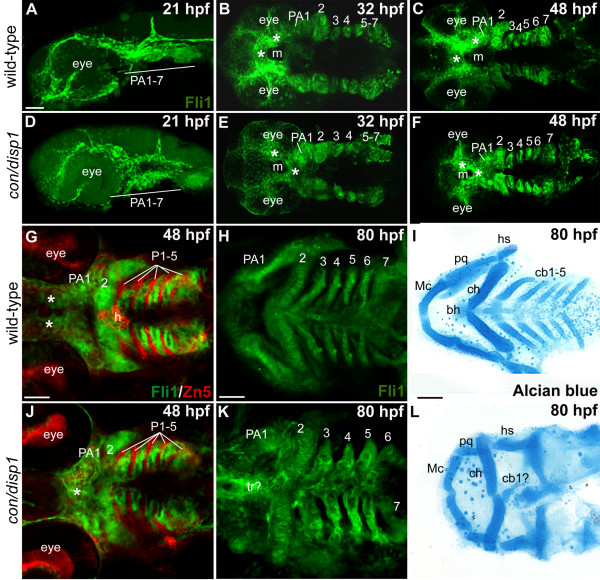
**CNCC patterning in the *con/disp1 *mutant is visualized by the *fli1gfp *transgene**. Lateral views (A,D) or ventral views (B,C,E-L) of confocal stack projections of *fli1*GFP^+ ^CNCC in wild type and *con/disp1 *mutants (A-H, J,K) or Alcian blue-stained larvae to visualize cartilages (I,L). (G,J) Zn5 (red) staining to visualize endodermal pouches (P 1-5) *in fli1*GFP embryos. (**A,D**) At 21 hpf, wild type (A) and *con/disp1 *mutants (D) reveal similar patterns of postmigratory-CNCC. (**B,C**) 32 hpf ventral views of wild type embryos reveal CNC condensations in PA (B), and segmentation of CNCC by 48 hpf (C). (**E,F**) By 32 hpf, *con/disp1 *anterior-most CNCC (asterisks) become mispatterned, while CNCC within more PA 2-7 are correctly patterned (E) and segmented by 48 hpf (F). (**G,J**) CNCC in wild type (G) and *con/disp1 *mutants (J) are interdigitated properly in well-formed endodermal pouches 1-5 (P 1-5) (Zn5, red). (**H,K**) *con/disp1 *mutants show developed, segmented CNCC in posterior arches (K). (**I,L**) CNCC within *con/disp1 *mutant posterior arches fail to become cartilage. Scale bar: 50 μM.

We tracked late-migrating and postmigratory-CNCC by crossing *con/disp1*^+/- ^fish to a transgenic line in which the *fli1 *promoter drives GFP in CNCC (*fli1*GFP:*con/disp1*) [27]. By 21 hpf, *fli1*GFP:*con/disp1 *CNCC have completed migration into the arch primordia and are broadly distributed in the future jaw and gills, similar to their *fli1*GFP:wild type siblings (Figure 2A, D). By 32 hpf, interactions between the post-migratory CNCC and surrounding epithelia take place to sequentially pattern the CNCC within the seven PA (PA1-7). Ventral views at this stage revealed patterning differences along the anterioposterior (AP) and mediolateral (ML) axis' in *fli1*GFP:*con/disp1 *mutants (Figure 2B, E). In wild type embryos, the anterior-most CNCC population migrated medial to the eyefield and resided anterior to the stomodeum (presumptive mouth, m) (Figure 2B). Contrarily, upon migrating between the eyefield, the anterior-most CNCC in *fli1*GFP:*con/disp1 *consistently resided posterior to the stomodeum, where they remained aberrantly patterned in mutant embryos at 48 hpf (anterior-most CNCC population denoted by asterisks, Figure 2B, C, E, F), similar to what has been shown for CNCC contributing to the dorsal neurocranium [18,23]. Previous fate-mapping studies of CNCC have shown that the anterior-most CNCC populations contribute to the dorsal neurocranium and mandibular arch (PA1) cartilages [18,41]. This suggests that the mispatterned population of post-migratory CNCC in *fli1*GFP:*con/disp1 *are at least partly fated to become the ventral jaw (Mc and pq) cartilages and thus may likely account for the later patterning defects seen in Alcian blue-stained larvae.

*fli1*GFP:*con/disp1 *CNCC patterning within PA2 was basically similar to wild type embryos (Figure 2B-K), with the exception that they were located slightly more medial than those in wild type embryos (Figure 2C-J). Viewing *fli1*GFP:*con/disp1 *at later stages (Figure 2H, K) revealed that *con/disp1 *CNCC resided in PA2, yet overall they were less organized and may have defects in stacking.

Interestingly, despite the complete absence of *con/disp1 *posterior arch cartilages, we detected no significant disruptions to CNCC patterning within *con/disp1 *mutant PA3-7 (Figure 2B-K). Further, by 48 hpf we clearly visualized demarcations between *fli1*GFP:*con/disp1 *GFP^+ ^CNCC and presumptive GFP^- ^endodermal cells (dark) upon which the CNCC that have migrated into PA2-7 condense, indicating proper arch segmentation (Figure 2C, F). By staining embryos with the endodermal pouch-labeling Zn5 antibody [42,43], we confirmed that GFP^- ^cells were endodermal and *con/disp1 *pharyngeal pouches were capable of forming (Figure 2G, J). Furthermore, *fli1*GFP:*con/disp1 *CNCC continued to express the *fli1*GFP-transgene and remained properly interdigitated within pharyngeal pouches up to 80 hpf (Figure 2H, K). Despite this, 80 hpf Alcian blue staining revealed that CNCC within the *fli1*GFP *con/disp1 *posterior arches do not differentiate into cartilage (Figure 2I, L). Taken in full, this data reveals that only the subset of CNCC that have migrated into the PA1 primordium rely on Hh-signaling to become properly patterned. Alternatively, in our hypomorphic allele, it is possible that this CNCC subset is simply the most sensitive to Hh-attenuation and larger disruptions would also affect CNCC patterning in PA2-7 as this phenotype has not been studied in more severely affected alleles.

### Pharyngeal arch chondrocyte differentiation is perturbed in *con/disp1 *mutants

To investigate the role of Hh-signaling in cartilage development we examined molecular markers that control chondrogenic and osteogenic differentiation in *con/disp1 *mutants. We studied the expression patterns of *sox9a *and *sox9b*, two transcription factors necessary for chondrogenesis [13,44], *runx2b*, a marker of chondrocyte and osteogenic differentiation [45,46], and *type II collagen *(*col2a1*), the predominant extracellular matrix protein in cartilage [47,48].

**Figure 3:**
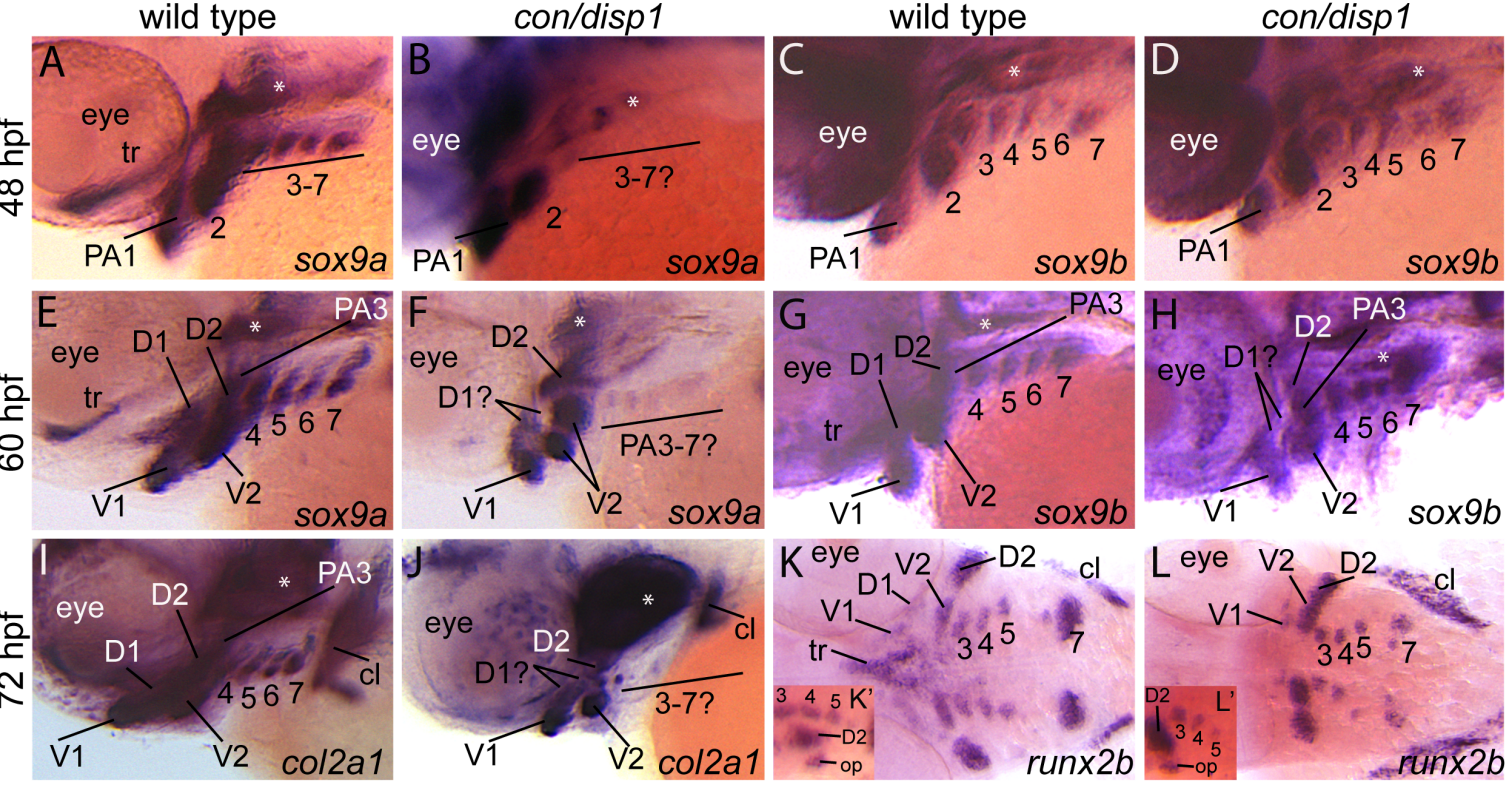
**Chondrogenic differentiation in *con/disp1 *CNCC**. Lateral views of 48 hpf (A-D), 60 hpf (E-H), 72 hpf (I,J) or ventral views of 72 hpf (K,L) wild type (A,C,E,G,I,K) or *con/disp1 *(B,D,F,H,J,L) embryos labeled with RNA probe for *sox9a *(A,B,E,F), *sox9b *(C,D,G,H), *col2a1 *(I,J) or *runx2b *(K,L). (**A,C**) *sox9a *marks CNCC in PA1-7 at 48 hpf in wild type embryos (A), however expression is lost in the posterior arch-residing CNCC in *con/disp1 *mutants (E). (**C,D**) *sox9b *continues to be expressed in 48 hpf PA1-7 condensations in wild type (B) and *con/disp1 *mutants alike (D). (**E-H**) At 60 hpf, *sox9a *and *sox9b *becomes downregulated in the dorsal subset of *con/disp1 *CNCC in the anterior arches (D1 and D2). *sox9a *remains absent in posterior arches in *con/disp1 *mutants, while *sox9b *remains robustly expressed in posterior arches. (**I,J**) At 72 hpf, *col2a1 *is reduced in *con/disp1 *D1 and D2 condensations and absent in PA3-7. (**K,L**) At 72 hpf, *runx2b *is absent in *con/disp1 *D1 and dorsal trabeculae precursors, but is present in PA condensation at wild type levels. (K',L') insets are of lateral views of same embryo shown in (K) and (L) respectively. Lateral views show presence of *runx2b *expression in opercle condensation in *con/disp1 *and wild type embryos alike. Asterisks indicate expression in mesoderm-derived polar cartilages. Images are at same magnification.

At 24 hpf, *sox9a *and *sox9b *expression marked four CNCC mesenchymal condensations (first three condensations are within PA 1-3 respectively, while the fourth condensation will subsequently become organized within PA 4-7) in *con/disp1 *mutants and wild type siblings alike (*sox9a *expression shown in additional file 2; *sox9b *expression data not shown). This indicated that both genes become properly induced in *con/disp1 *CNCC. However, by 48 hpf the *sox9a *transcript was absent in condensations within PA3-7 in *con/disp1 *mutants (Figure 3B). In contrast, *sox9a *expression remained detectable in PA 1-2-residing condensations in *con/disp1*mutants (Figure 3B). Despite the gene expression reductions of *sox9a*, *sox9b *expression at 48 hpf yielded no detectable differences at this stage between *con/disp1 *and their wild type siblings (Figure 3C, D).

By 60 hpf, CNCC in the first two arches became organized into dorsal and ventral prechondrogenic cartilage condensations, which can be visualized by *sox9a *and *sox9b *gene expression (Figure 3E, G). It has been suggested that organization of CNCC into these dorsoventral (DV) domains likely precedes their later chondrification into dorsal (pq, ptp, hs) and ventral (Mc and ch) cartilage structures [49,50]. At this stage, we detected a reduction in the number of *sox9a *and *sox9b *expressing cells residing in dorsal domains of PA1-2 in *con/disp1 *mutants compared to wild type siblings (Figure 3E-H). Despite this, expression of both genes was retained at normal levels in CNCC residing within the PA1-2 ventral domains (Figure 3E-H). Expression of the *sox9 *genes in the posterior arches at 60 hpf revealed that *con/disp1 *CNCC in these PA continued to express *sox9b*, while *sox9a *remained absent in these cells (Figure 3E-H).

*col2a1 *is essential for chondrogenesis [47,48], and is believed to be dependent on *sox9a *in CNCC [49]. Consistent with this, *col2a1 *expression was absent within *con/disp1 *posterior arch-residing CNCC at 72 hpf, while remaining robustly expressed in the ventral domains of anterior arches (Figure 3I, J)

To further study the role of Hh-signaling in chondrocyte development we assessed *runx2b *expression in *con/disp1 *mutants. Mammalian Runx2 genes act as major regulators for osteoblastogenesis [51]. *runx2b *is expressed in mesenchymal condensations within the PA at 2-3 dpf in zebrafish and controls chondrocyte differentiation [45,46]. At 72 hpf, we found the gene transcript was present in *con/disp1 *mesenchyme within each PA condensation, albeit at a reduced level in PA1 (Figure 3K, L). Additionally, *run2b *was robustly expressed in the opercle (Figure 3K', L' insets) and cleithrum tissue groups in *con/disp1 *mutants, while its expression was consistently absent in *con/disp1 *dorsal neurocranial precursor cells (Figure 3K, L). Thus, *runx2b *expression in PA mesenchyme does not appear to be directly dependent on Hh-signaling.

In full, this expression data suggests that the Hh-pathway likely controls chondrocyte development in the posterior PA through the transcription factor *sox9a*, although the pathway may be required for broader transcription factor expression in the dorsal jaw precursors.

### Disp1 is differentially required for postmigratory-CNCC development gene expression

**Figure 4:**
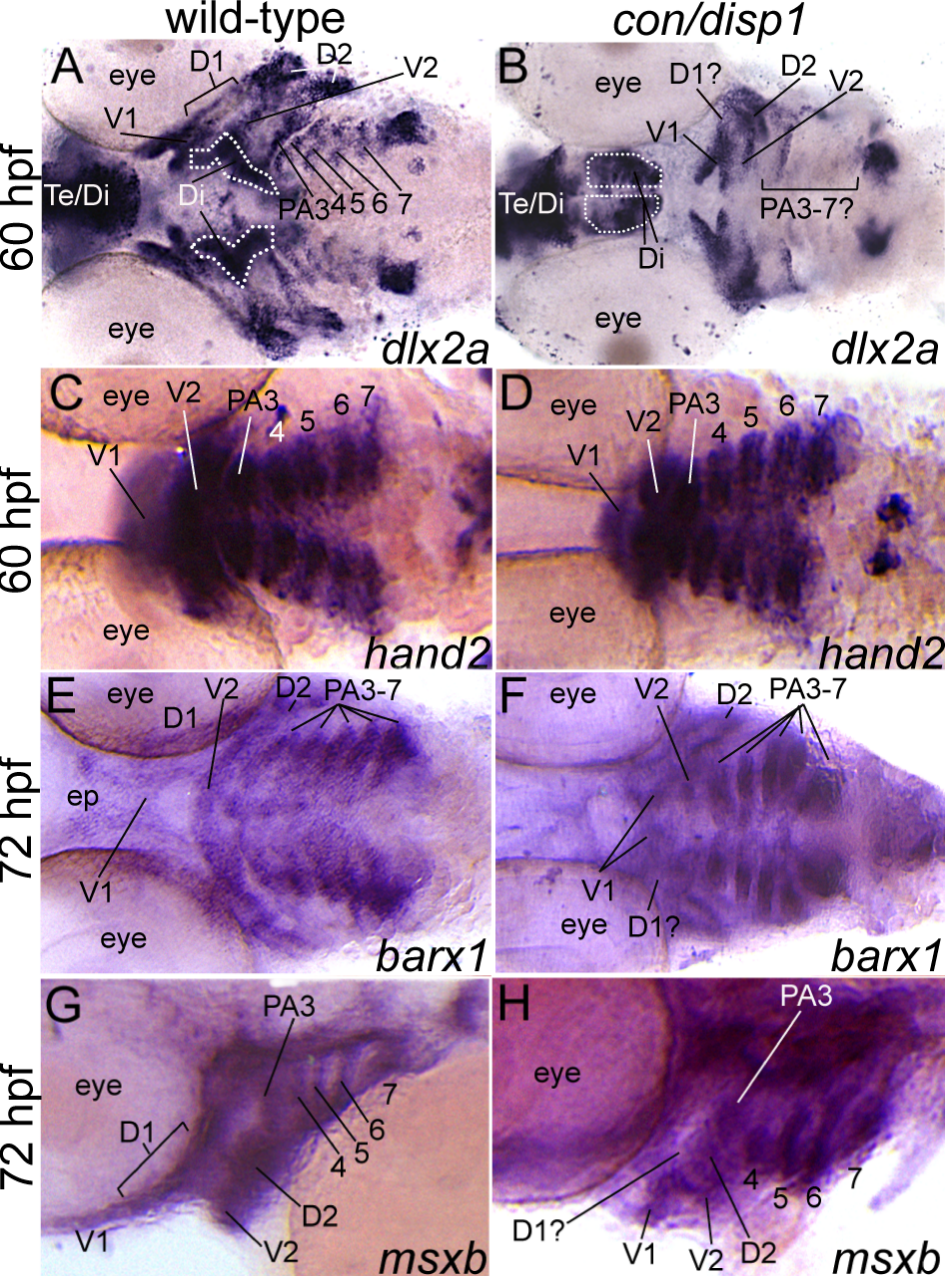
***con/disp1 *mutants show variable postmigratory-CNCC gene expression defects**. Ventral, 60 hpf (A-D), ventral, 72 hpf (E,F) or lateral, 72 hpf (G,H) views of wild type (A,C,E,G) or *con/disp1 *(B,D,F,H) embryos labeled with RNA probe for *dlx2a *(A,B), *hand2 *(C,D), *barx1 *(E,F), *msxb *(G,H). (**A,B**) *dlx2a *is downregulated in dorsal domain of PA1 (D1) and posterior arches in *con/disp1 *mutants. (**C,D**) *hand2 *is expressed in CNCC within each *con/disp1 *PA. (**E,F**) At 72 hpf, *barx1 *expression marks the anterior and posterior arches in wild type embryos (E), but is downregulated in the *con/disp1 *D1 domain of PA1 (F). (**G,H**) *msxb *is downregulated in the D1 domain, but is expressed at wild type levels in posterior arch-residing CNCC. Scale bar: 50 μM.

To continue to study the role of Hh-signaling in ventral cartilage development, we next sought to determine the expression of the essential neural crest differentiation markers *dlx2a, hand2, barx1*, *msxb *and *msxe *genes, which are expressed in postmigratory-CNCC and whose homologs are required for proper mammalian PA development [52-59]. Expression of all of these genes at 48 hpf, or earlier, in *con/disp1 *mutants was indistinguishable from wild type siblings (data not shown). However, by 60 hpf we detected a reduction in the number of *dlx2a*^+ ^cells residing in the *con/disp1 *anterior arches (PA1-2), possibly corresponding to the dorsal prechondrogenic condensations (D1 and D2), although it was difficult to specifically detect which anterior arch had reduced *dlx2a *staining within in the *con/disp1 *mutant (Figure 4A, B). Furthermore, we clearly noticed a significant reduction in *dlx2a *gene expression in the *con/disp1 *posterior arches (Figure 4A, B). Thus, as is the case for *sox9a *expression (Figure 3), Hh-signaling is required to maintain *dlx2a *expression in the CNCC residing in PA3-7. Additionally at 60 hpf, *dlx2a *was also detectable in the diencephalon (dotted lines are shown to outline the ventral diencephalon). Simultaneous visualization of brain and CNCC by *dlx2a *staining in *con/disp1 *embryos further underscored the AP and ML patterning defects of CNCC within the PA of these mutants (Figure 4A, B). Unlike *dlx2a*, the remaining genes studied were maintained in *con/disp1 *CNCC mesenchyme (Figure 4C-H and *msxe *expression data not shown), despite being slightly downregulated in the dorsal domains of the hypoplastic PA1 and PA2, suggesting that these genes may be regulated independently of Hh-signaling in the PA.

### Fate of CNCC within the posterior arches in *con/disp1 *mutants

The persistence of general neural crest markers in the *con/disp1 *mesenchymal condensations within the posterior arches (PA3-7) suggests that CNCC survive in these arches. This hypothesis is consistent with our findings, and those by Eberhart et al., 2006 and Wada et al., 2005, which revealed that cell proliferation and cell death levels within the facial primordia of Hh-mutants (severe loss-of-function as well as hypomorphic alleles) are no different than wild type siblings [18,23]. Despite these three corroborating reports, a separate study by Teraoka et al., 2006 attributed zebrafish jaw outgrowth defects in Cya treated animals to changes in cell death and cell proliferation, which they observed at 65 hpf [60]. Our exhaustive efforts to show differences in cell death (additional file 3) and cell proliferation in the *con/disp1 *mutant at stages precluding, preceding or including this timepoint were unable to find any significant changes between *con/disp1 *mutants and wild type embryos.

**Figure 5:**
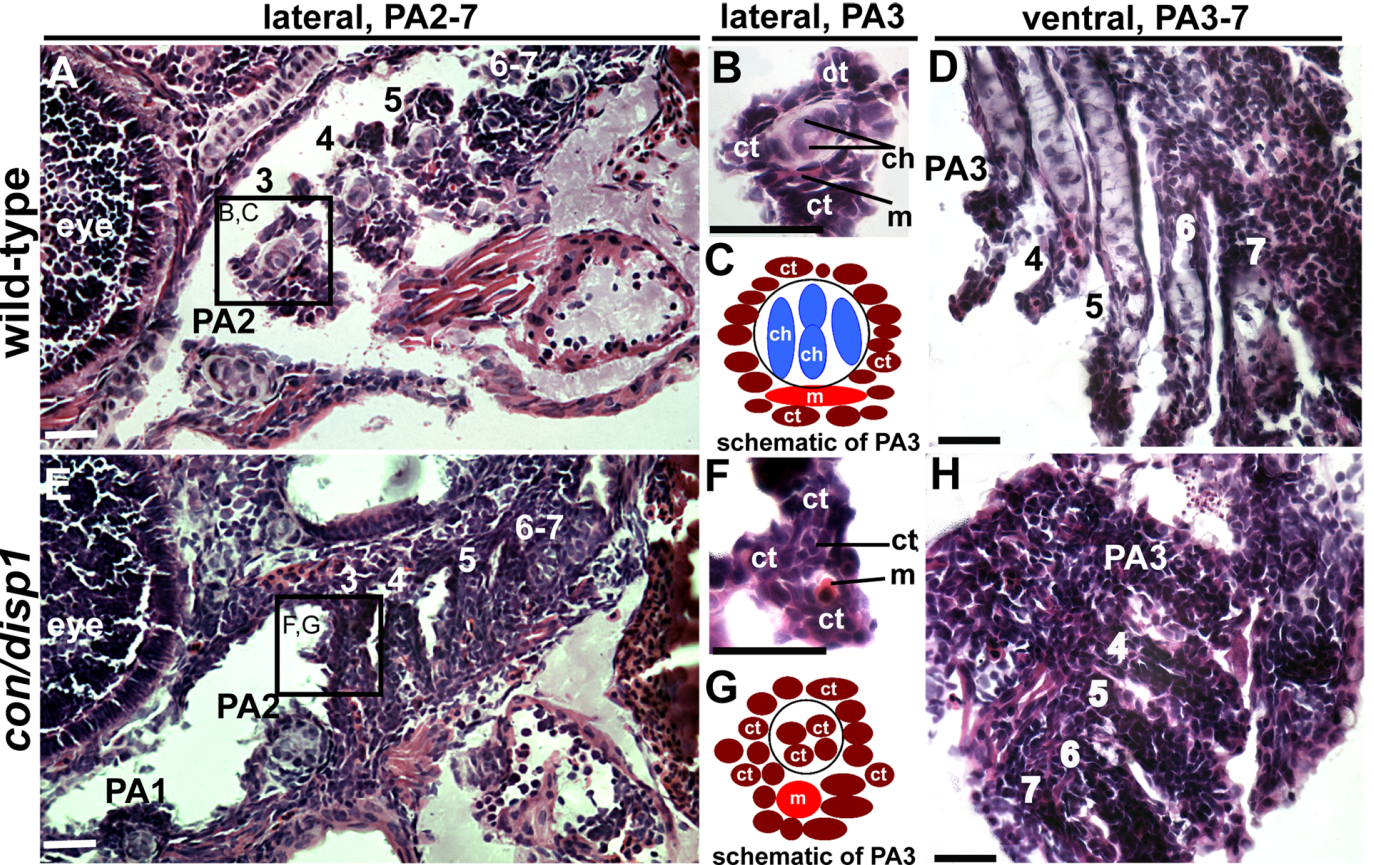
***con/disp1 *posterior arch-residing CNCC become fibrous-connective tissue**. Lateral, (A,B,E,F) or ventral (D,H) H&E stained 10 μM sections of 5 dpf wild type (A,B,D) or *con/disp1 *mutant (E,F,H) larvae. (**A,C**) Chondrocytes are surrounded by connective tissue within each wild type PA (A), with higher magnification of PA3 revealing individual cell types (B). Box in (A) surrounding PA3 is viewed at higher magnification in (B). (**E,F**) *con/disp1 *mutants lack chondroctyes in PA3-7 (E), higher magnification of PA3 displays ectopic fibrous-connective tissue in PA3 (F). Box in (E) is viewed at higher magnification in (F). (**C,G**) Schematic of cell types visualized in wild type (C) and *con/disp1 *(G) PA3. (**D,H**) Horizontal sections show chondrocytes stacked within wild type posterior arches (D), while fibrous-connective tissue populate the *con/disp1 *posterior arches (H). Scale bar: 50 μM.

An alternative hypothesis to CNCC failing to proliferate or survive in the PA and thus accounting for reduced craniofacial cartilage, could be that CNCC persist in the PA, but do so as a non-cartilage CNCC derivative. Mesenchymal CNCC that have migrated into the PA primordia are multipotent, able to give rise to fibrous-connective tissue such as smooth muscle cells and myofibroblasts, in addition to specialized-connective tissue such as facial cartilage [61]. Therefore, in the absence of pro-chondrogenic gene expression (*sox9a*, *col2a1*, *dlx2a*) we wondered if the persisting posterior arch-residing CNCC gave rise to fibrous-connective tissue or an alternative cell type. To address this, we stained 5 day-old wild type and *con/disp1 *larvae with hematoxylin and eosin in order to differentiate cell types. Wild type larvae revealed the presence of chondrocytes (bluish-white staining, large vacuolated cell) in each PA (Figure 5A). Viewing PA3 at higher magnification revealed that chondroctyes were neatly arranged into a circular rod, lying dorsal to the arch's muscle cell (red), and surrounded by a mass of fibrous-connective tissue (dark staining, smaller cell) (Figure 5B). Within *con/disp1 *larvae, chondrocytes were clearly present within the hyoid arch, however chondrocytes were not detectable in the posterior arches (Figure 5E). Rather, these arches were populated fully by fibrous-connective tissue (Figure 5E). Similar to the wild type PA3 organization, *con/disp1 *PA3 ectopic fibrous-connective tissue cells were organized within a small circular rod, lying just dorsal to the arch's muscle cell, surrounded by connective tissue in a highly ordered fashion (Figure 5B, F and cellular organization is schematized in Figure 5C, G). Horizontal sections through the *con/disp1 *PA confirmed the widespread presence of fibrous-connective tissue within PA3-7 (Figure 5D, H). Thus, in the absence of Hh-signaling CNCC in the posterior arches become, or alternatively remain, fibrous-connective tissue cells. This may potentially explain why this study and others have not found an increased number of dying cells in the heads of Hh-signaling mutants despite the lack of cartilage elements in the PA (our study) or the neurocranium [18,23] of these animals.

### Hh-signaling is required for cranial joint formation

**Figure 6:**
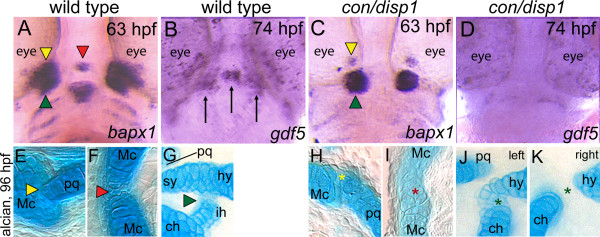
**Joint defects are apparent in *con/disp1 *mutants**. Ventral views (A-D) of wild-type (A,B) or *con/disp1 *mutants (C,D) labeled with RNA probe for *bapx1 *at 63 hpf (A,C) or *gdf5 *at 74 hpf (B,D). Alcian blue-stained cartilage at 96 hpf in wild-type (E-G) or *con/disp1 *mutant (H-K) embryos. (**A,C**) *bapx1 *prefigures bilateral and midline joints in wild-type embryos, but expression is reduced bilaterally and absent in the midline of *con/disp1 *mutants. Different color arrowheads used to denote individual cranial joints in A,C and E-G. Asterisks of the same color designate a reduced joint in *con/disp1 *mutants in H-K. (**B,D**) *gdf5 *is strongly expressed bilaterally in the first arch joint region (arrows) and in a group of cells in the midline that prefigures the basihyal cartilage element (arrow) (B). Expression of *gdf5 *is significantly reduced in both bilateral and midline domains in *con/disp1 *mutants (D). (**E-G**) In wild-type embryos, bilateral joints form between the Mc and pq (E, yellow arrowhead) and hs and ch (G, green arrowhead), as well as at the midline between the bilaterally formed Mc (F, red arrowhead). (**H-K**) In *con/disp1 *mutants, a poorly formed joint between the Mc, which fails to extend into a RAP element, and a severely reduced pq is visualized (H, yellow asterisk). In the second arch, we see either ectopic cartilage cells at the joint site between the hs and ch elements (J, green asterisk) or a failure of the hs and ch to join (K, green asterisk). Both phenotypes are occasionally present in the same larvae (left designates left side, right designates right side of single larvae). Further, the bilateral mc elements fuse at the midline to disrupt the joint (I, red asterisk).

Our analysis of the mandibular and hyoid arch cartilages in *con/disp1 *mutants revealed abnormal joints (Figure 1C-F). By 96 hpf, bilateral joints were present between the ventral Mc and dorsal pq of the mandibular arch (Figure 6E) and the ventral ch and the dorsal hs of the hyoid arch (Figure 6G). Frequently in the *con/disp1 *mutant, the cartilaginous retroarticular process (RAP) that forms at the posterior extent of the Mc element was reduced and the jaw joint between the Mc and pq was difficult to visualize (Figure 6E, H). In the *con/disp1 *mutant hyoid arch, ectopic cartilage cells were often present between the ventral ch and dorsal hyomandibular (hy) cartilage elements (Figure 6J), where localized inhibition of chondrogenesis at the joint site normally produces a thin interhyal (ih) element (Figure 6G). Less frequently, dorsal and ventral PA2 cartilage elements failed to articulate with one another (Figure 6K). Furthermore, midline joint formation was also disrupted in *con/disp1 *mutants, whereas ectopic cartilage condensed at the presumptive mandibular arch joint domain between bilateral Mc elements (Figure 6F, I).

To provide a potential genetic mechanism for the role of Hh-signaling in cranial joint formation we studied the expression of the *bagpipe*-related transcription factor *bapx1 *and the TGFβ signaling molecule *gdf5*, both of which are expressed in early cartilage condensations and are required for vertebrate joint organization and growth [62-67]. At 63 hpf, ventral views of *con/disp1 *mutants revealed the presence of two bilateral *bapx1 *condensations that prefigure the mandibular and hyoid arch lateral joints (Figure 6A). However, when compared to wild type siblings the numbers of *bapx1*^+ ^cells in these bilateral domains were reduced (Figure 6C). Those remaining *bapx1*-expressing cells may reside in the PA1 joint region where the Mc and pq elements attempt to set up a joint between the poorly developed cartilage elements (Figure 6H). Furthermore, *bapx1 *expression was completely reduced within the midline of the *con/disp1 *mutant (Figure 6C). At 74 hpf, *gdf5 *expression can be detected in a bilateral population of cells that prefigures the jaw joint, as well as in a cell population at the midline that prefigures the unpaired ventral basihyal (Figure 6B). However, these expression domains are significantly reduced in *con/disp1 *mutants, correlating with the reduced PA1 midline joint and basihyal cartilage phenotype (Figure 6I and Figure 1D). Together this data reveals novel requirements for Hh-signaling in the development of jaw joints, organization of the first arch RAP cartilage element and the establishment of jaw joint domains during the second day of development, which may be partly attributed to a reduction in early gene expression of two genes, *bapx1 *and *gdf5*. This phenotype is likely hidden in many Hh-signaling mutants due to the additional requirement for Hh-signaling on anterior arch chondrogenesis near the joint regions (Figure 6K, findings in this study and [18]) which in more severely affected larvae leads to the loss of all cranial skeletal elements [14].

### *disp1 *and *shh *are co-expressed in brain and facial epithelia during development

**Figure 7:**
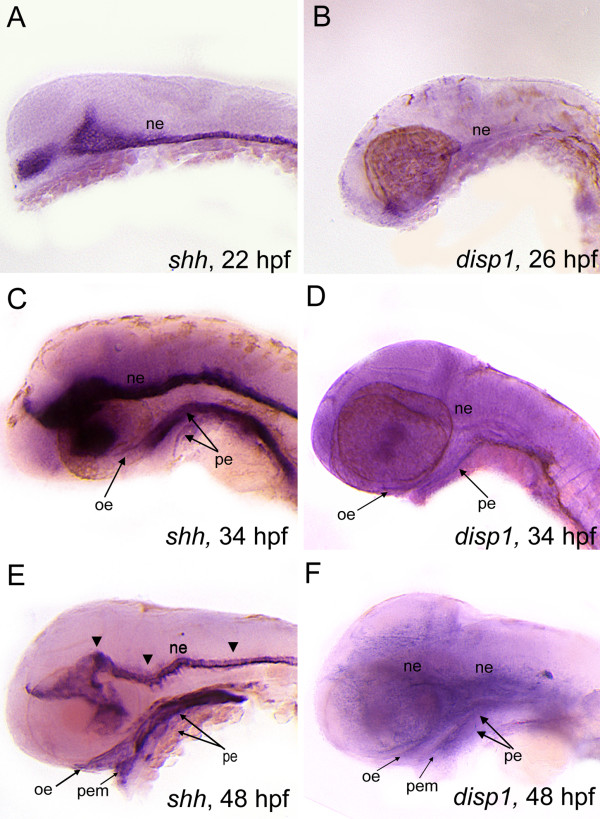
***shh *and *disp1 *are coexpressedin the developing head**. Lateral views of wild type embryos labeled with RNA probe for *shh *(A,C,E) or *disp1 *(B,D,F). (**A,B**) At 22 hpf (*shh*) - 25 hpf (*disp1*)are both expressed in ventral neuroectoderm (ne). (**C,D**) At 34 hpf, *shh *and *disp1 *expression becomes detectable in oral ectoderm (oe) and pharyngeal endoderm (pe), in addition to the neuroectoderm (ne). Arrowheads denote expression within the neuroectoderm for both *shh *and *disp1*. (**E-F**) By 48 hpf, *shh *and *disp1 *expression becomes more prominent in the oe and pe, and is further expanded to the pharyngeal ectodermal margin (pem). *shh *and *disp1 *expression persists in the ne at 48 hpf.

To determine the cells that produce and secrete Hh-ligands during craniofacial development we investigated the expression patterns for *disp1 *and *shh*. We confirmed that both genes are co-expressed within the dorsal mesoderm during gastrulation and persist in their notochord and presomitic-mesoderm derivatives during somitogenesis [26,68]. By 22-26 hpf both genes are localized to the ventral brain (neuroectoderm, ne) (Figure 7A, B). By 34 hpf, *shh *expression is expanded to the oral ectoderm (oe) and the pharyngeal endoderm (pe) and by 48 hpf we also could visualize robust expression in the pharyngeal ectodermal margin (pem) (Figure 7C, E) where *shh *remained up to 72 hpf (data not shown). *disp1 *RNA transcripts appeared to be expressed in a similar spatiotemporal fashion to *shh *in neural and facial tissues (Figure 7D, F), although specific tissue detection for this RNA probe was made difficult due to a high signal-to-noise ratio in embryos older than 30 hpf. Despite this limitation, and based on the conserved functional role for Disp1 in Hh-expressing cells and our visualization of *disp1 *transcripts in facial tissue beginning at 34 hpf, we strongly believe that *disp1 *is localized to the oe, pem and pe tissues by 2 days post fertilization.

### Hh-signaling is temporally required early and late to direct ventral cartilage development

**Figure 8:**
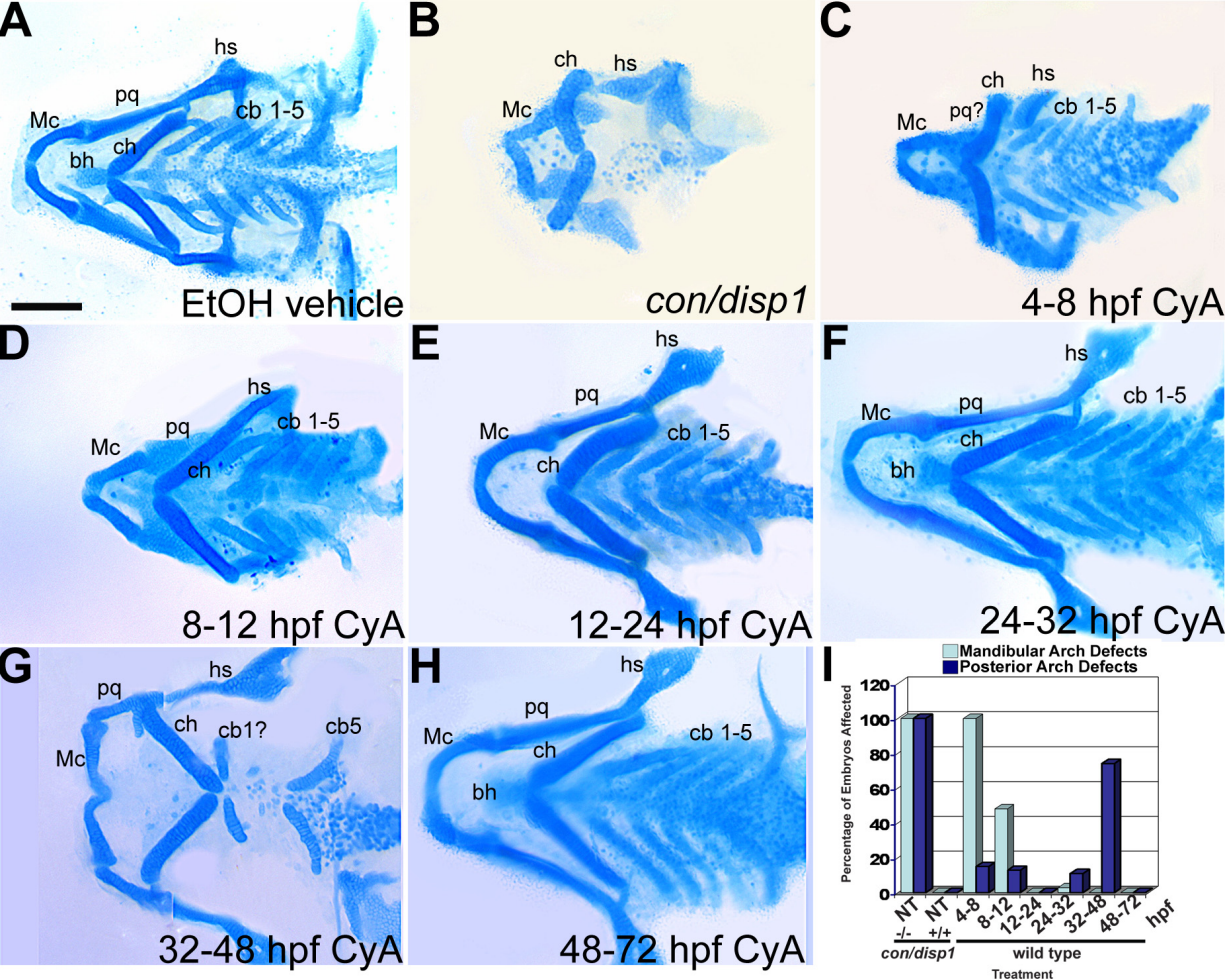
**Early and late Cyclopamine treatments disrupt ventral PA development**. (A-H) 96 hpf Alcian blue-stained cartilage. Wild type treated with ethanol (EtOH) vehicle (4-48 hpf) (A), untreated *con/disp1 *mutant (B) and wild type treated with 100 μM Cya at stages between 4-72 hpf (C-H). (**B,C**) 4-8 hpf treatment causes reduced jaw cartilage and outgrowth defects (C), similar to *con/disp1 *mutant (B), while hyoid and posterior arch cartilages are unaffected (C). (**D-F**) 8-32 hpf treatments had little effect on PA cartilage development, with occasional jaw cartilage reductions in 8-12 hpf treatment (D). (**G**) 32-48 hpf treatment eliminated most cb cartilages, without reducing anterior arch cartilage. (**H**) Treatments after 48 hpf have no effect on PA cartilage development. (**I**) Mandibular arch patterning defects and posterior arch chondrogenesis defects are summarized for each treatment (n = 20 embryos per treatment). Scale bar: 50 μM.

The dynamic expression pattern of *shh*, first in the brain and then in facial epithelia, coupled with previous findings from Wada et al., 2005 that Hh-signaling controls dorsal neurocranium chondrogenesis at timepoints from gastrulation up to 2 days post fertilization, prompted us to study the temporal requirement for Hh-signaling in PA skeleton development. We treated wild type embryos with Cya, to antagonize Hh-signaling activation by compromising Smo action [69,70], at different developmental stages from 4-72 hpf. A 100 μM Cya concentration was chosen as this concentration has been used by our laboratory and others to effectively block Hh-signaling sensitive *ptc1 *or *gli1 *expression [71,72]. Embryos treated during gastrulation (4-8 hpf), then allowed to develop to 96 hpf for cartilage analysis by Alcian blue staining, exhibited a general decrease in Mc and pq chondrogenesis and jaw outgrowth when compared to vehicle-treated control embryos alone (Figure 8A, C). The mandibular arch cartilage defects of gastrulation-stage treated embryos were similar to those described for the *con/disp1 *mutant (Figure 8B). Despite this, hyoid and posterior arch cartilages were only slightly reduced and fairly well patterned in gastrulation stage treated embryos (Figure 8C). Cya-treatments administered during stages of early neurulation and neural crest specification (8-12 hpf) occasionally resulted in reduced mandibular arch cartilages but rarely affected other PA cartilages (Figure 8D). Next, we found that in contrast to earlier treatments, Cya-treatments during CNCC migration (12-24 hpf) or during the timepoint critical for CNCC condensation within facial epithelial (24-32 hpf) produced no negative effects on the development of the PA elements (Figure 8E, F). We next treated embryos with Cya during the late pharyngula stage (32-48 hpf). Strikingly, embryos treated during this stage exhibited severe cb cartilage loss in the posterior arches (Figure 8G). Despite this, Cya treatment during the late pharyngula stage had only minor anterior arch deficits, including inverted Mc and ch elements, a common developmental by-product in the PA when the gill cartilages are absent [34]. Finally, blocking Hh-signaling in embryos after 48 hpf had no influence on PA cartilage development (Figure 8H). Collectively, this data indicates two critical time periods during which Hh-signaling is required for PA cartilage development, one early (4-8 hpf, gastrulation) to direct jaw outgrowth and chondrogenesis within portions of the anterior arches, and one later (32-48 hpf, late pharyngula stage) to promote chondrogenesis in the posterior arches (summarized in Figure 8I).

### Early and late Hedgehog inhibition influences CNCC development

**Figure 9:**
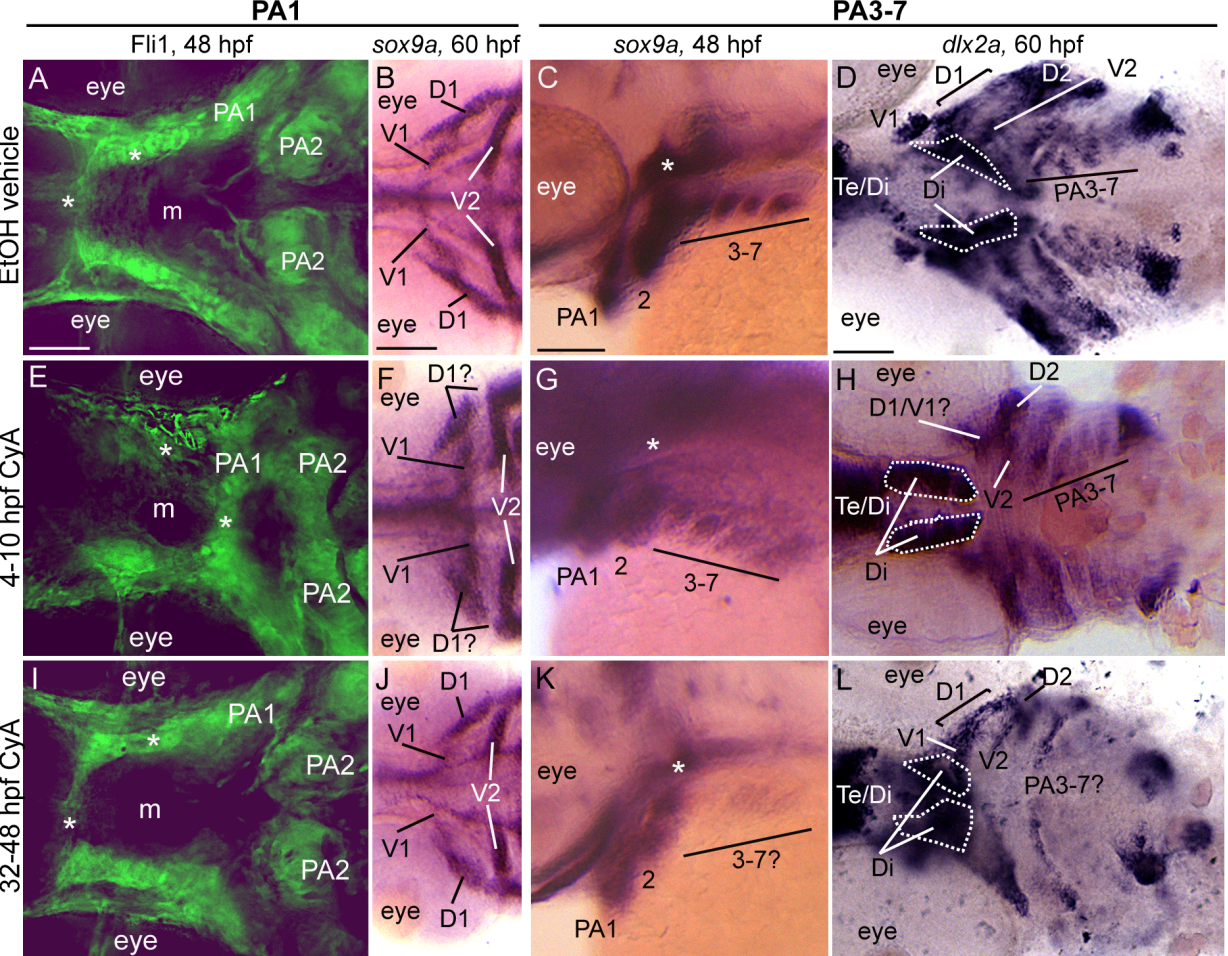
**Early and late Cyclopamine treatments lead to CNCC defects**. Ventral (A,B,D,E,F,H,I,J,L) or lateral (C,G,K) views at 48 hpf (A,C,E,G,I,K) or 60 hpf (B,D,F,H,J,L) of vehicle-treated wild type (A-D) or Cya-treated wild type (E-L) embryos that are Fli1gfp positive (A,E,I) or labeled with RNA probe for *sox9a *(B,C,F,G,J,K) or *dlx2a *(D,H,L). (**A,E,I**) By 48 hpf, vehicle-treated wild type embryos (A) display proper patterning of anteriormost CNCC (asterisks), as do 32-48 hpf Cya-treated embryos (I); however, 4-10 hpf Cya-treated embryos (E) show mispatterning of anteriormost CNCC. (**B,F,J**) Vehicle treated embryos display normal *sox9a *expression in the dorsal (D1) and ventral (V1) CNCC condensations in the first arch (B), as do 32-48 hpf Cya-treated embryos (J). In 4-10 hpf Cya-treated embryos, ventral (V1) CNCC condensations maintain *sox9a *expression, while *sox9a *is greatly reduced in the dorsal (D1) region of the first arch. (**C,D,G,H,K,L**) Treating wild type embryos with vehicle or Cya from 4-10 hpf does not influence *sox9a *expression at 48 hpf(C,G) or *dlx2a *at 60 hpf (D,H) within posterior arch residing CNCC, while treating wild type embryos with Cya at 32-48 hpf leads to reduction in *sox9a *gene expression at 48 hpf (K) and *dlx2a *at 60 hpf (L) within CNCC in the posterior arches. Asterisks in (C,G,K) indicate *sox9a *expression in mesoderm-derived polar cartilages. Scale bar: 50 μM.

In light of our Cya results on ventral skeleton development, we wondered if Cya treatments during gastrulation and the late pharyngula stage could together explain the CNCC defects we identified in *con/disp1 *mutants. We found that treating embryos with Cya during gastrulation (4-10 hpf) sufficiently and reproducibly disrupted the patterning of PA1-localized *fli1:gfp*^+^CNCC at 48 hpf (9/10 treated embryos displaying patterning defect in Figure 9E), while vehicle-treated siblings never displayed patterning defects (0/9 treated embryos displayed defect, Figure 9A). Mispatterned CNCC in PA1 were aberrantly localized posterior to the eyefield/presumptive mouth, similar to *con/disp1 *mutants (Figure 2F). Later treatments, notably during the late pharyngula stage (32-48 hpf), did not result in PA1 CNCC patterning defects (0/10 treated embryos displayed defect, Figure 9I). Thus, the crucial time for Hh-signaling in promoting proper patterning of CNCC within PA1 is during gastrulation (our studies, [18]). Along with PA1 patterning defects, we found that *sox9a *expression was significantly reduced in the PA1 dorsal domain (D1) at 60 hpf in embryos treated with Cya during gastrulation stages (8/15 treated embryos displaying expression defect, Figure 9F), correlating with the reduced dorsal cartilages in the anterior arches (Figure 8C). Despite reduced *sox9a *gene reduction in the dorsal first arch domain, gastrulation-stage treated embryos displayed normal *sox9a *expression in the ventral domain (presumptive Mc) as well as small subset of dorsal-lying cells, likely correlating to the remaining pq cartilage elements (Figure 8C), similar to the *con/disp1 *mutant cartilage phenotype (Figure 1D). Unlike gastrulation-stage treated embryos, vehicle-treated siblings displayed normal *sox9a *expression in the PA1 (0/10 treated embryos displayed defect, Figure 9B), as did siblings treated after gastrulation, including the late pharyngula stage (0/15 treated embryos displayed defect, Figure 9J).

As shown previously, by 48 hpf, *con/disp1 *CNCC in posterior arches no longer express *sox9a*, and *dlx2a *expression is significantly downregulated in the same cells by 60 hpf. We found that Cya-treatment during the late pharyngula stage eliminated *sox9a *expression (9/12 treated embryos displayed defect, Figure 9K) in mesenchymal condensations within the posterior arches at 48 hpf, and *dlx2a *by 60 hpf (8/10 treated embryos displayed defect, Figure 9L). In contrast, vehicle treatments never influenced gene expression in the posterior arches (0/10 treated embryos for *sox9a *and 0/10 treated embryos for *dlx2a *displayed defects, Figure 9C, D), nor rarely did any earlier Cya-treatments, including during gastrulation (1/11 treated embryos for *sox9a*, 0/10 treated embryos for *dlx2a *displayed defects, Figure 9G, H). In our *con/disp1 *studies above we showed that *sox9b *and *barx1 *are regulated independently of Hh-signaling in the majority of PA-derived mesenchyme (an exception is *sox9b *in dorsal subset of anterior arch mesenchyme, see Figures 3 and 4). Similarly, *sox9b *and *barx1 *expression in mesenchymal condensations within the PA were not influenced by Cya treatments during gastrulation or the late pharyngula stage (additional file 4).

Collectively, this data shows that CNCC patterning and chondrogenesis defects in the first arch of Hh-signaling mutants can be attributed to a loss of Hh-signaling during gastrulation stages (4-10 hpf). Moreover, the crucial time for Hh-signaling in promoting posterior arch chondrogenesis is the late pharyngula stage (32-48 hpf).

## Discussion

HPE is a common malformation of the face in humans. Its etiology is highly heterogeneous, likely due to the involvement of multiple genetic factors that are required in a variety of craniofacial tissues and at various times during facial growth and patterning [17,73]. Disruption of the Hh-signaling pathway is the most frequently reported deficit underlying HPE, or its closely related clinical microforms of the disorder, in humans [2]. For this reason, multiple studies have sought to experimentally demonstrate the requirements for the Hh-signaling pathway in craniofacial tissues and facial precursors. In the zebrafish, these previous studies have either focused solely on the development of the skull and upper jaw [18,23,33], or utilized severe loss-of-function alleles representing extreme cases of cranial skeleton loss [14]. Here we provide a detailed characterization for the role of Hh-signaling in the PA skeleton by utilizing a hypomorphic allele of Hh-signaling (*con/disp1*). Indeed, it has been suggested by others that these less-severe alleles may provide very useful insights into the etiology of Hh-associated HPE as they display characteristic craniofacial abnormalities in the absence of more debilitating phenotypes, such as cyclopia and nervous system deficits [2,74].

Our study revealed multiple requirements for Hh-signaling during CNCC development that influences the final facial skeletal pattern. First, Hh-signaling controls jaw outgrowth by influencing both patterning and chondrogenic differentiation of postmigratory-CNCC that reside in the anterior arches. Using the Hh-inhibitor Cya we revealed that Hh-signaling is needed during gastrulation for proper jaw formation. Hh-inhibition during gastrulation in zebrafish and chick embryos is also associated with cyclopia and mid-facial dysplasia (data not shown, [17]), and has also been shown to influence patterning and chondrogenesis in the zebrafish dorsal skeleton [18,23]. These phenotypes mimic severe loss-of-function mutations within human *SHH *in HPE, which is characterized by facial anomalies including cyclopia, cleft lip, and underdeveloped jaw [1]. Despite impairing jaw outgrowth, multiple cranial structures remained in the lower jaw of *con/disp1 *larvae. This not only revealed that some cranial structures were more sensitive to Hh-attenuation (i.e. the dorsal jaw and jaw support elements), but also allowed for detection of deficits in the jaw joint.

Our work further revealed a requirement for Hh-signaling in cartilage development within the posterior gill-forming PA. *con/disp1 *mutant posterior arch-residing CNCC fail to chondrify, despite becoming properly patterned within well-formed endodermal pouches. Cya treatments revealed the single and crucial time for Hh-signaling in this event is during the late-pharyngula stage, well beyond the time when CNCC have migrated into the PA primordia. It is not surprising that Hh-signaling is required by postmigratory-CNCC in the posterior arches as Hh-ligands become widely expressed throughout the facial epithelial tissues shortly after the time in which CNCC migrate into the face. By examining CNCC differentiation in these arches, we found that certain genes are specifically downregulated in the absence of Hh-signaling, and histological preparation of the gill arches suggest that CNCC are transfated to fibrous-connective tissue as a result. It is clear from these findings that multiple signaling interactions involving the Hh-pathway must occur for proper craniofacial development, and this likely reflects the variability in human phenotypes associated with disruptions to the pathway [1]

### Hh-signaling is required for mandibular arch patterning and chondrogenesis

Frontonasal development and the outgrowth of anterior craniofacial structures are strongly influenced by the facial oral ectoderm [21,23,75]. Fate mapping has revealed that dorsal CNCC precursors (neurocranium and pq) condense upon the oral ectoderm roof upon their arrival to the face, while ventral CNCC precursors (Mc and a subset of pq) migrate to, and condense upon, the oral ectoderm floor [18,41]. The ability of CNCC to condense upon the oral stomodeum is negatively affected by the loss of Hh-signaling [18], likely resulting in the aberrant localization of first arch CNCC. In our studies of *fli1*GFP:*con/disp1 *mutants, CNCC that have migrated into the first arch were conspicuously absent just anterior to the stomodeum, and rather these cells preferentially localized posterior to the stomodeum. Interestingly, the anterior-most CNCC in the *fli1*GFP:wild type siblings never localized posterior to the stomodeum, suggesting that Hh-signaling may normally act to restrict CNCC from this region, either through promoting CNCC-stomodeum adhesion and/or by inducing midline tissue expansion posterior to the stomodeum that would serve as a physical barrier.

In addition to patterning defects, our studies revealed cell differentiation defects in the anterior-most CNCC. Local morphogenetic signals from the oral ectoderm, such as *shh*, are likely involved in promoting mesenchymal differentiation and skeletal outgrowth in the jaw and neurocranium. In chick embryos, *Shh *from the oral ectoderm influences expression of critical CNCC markers to promote maxillary and frontonasal outgrowth [21,76,77]. By closely examining the cell differentiation defects in the *con/disp1 *mutants, we found significant variability in the expression of CNCC markers between mesenchymal condensations in different DV domains within the lower jaw (PA1-2). Notably, neural crest-specific gene expression often appeared to be reduced in *con/disp1 *dorsal condensations within anterior PA, while expression in ventral condensations were less affected. This is similar to the anterior craniofacial development of *Shh*^-/- ^mouse embryos wherein severe hypoplasia is seen in the proximal (dorsal) mandibular arch and maxillary mesenchyme, while the more distal (ventral) mandibular arch regions are less affected [78]. Furthermore, a shortened, hypoplastic ventral, first arch Mc element is the only remaining structure when Hh-responsiveness is specifically removed in all cranial precursors (CNCC) in the mouse [22]. Collectively, this suggests that the dorsal jaw, dorsal jaw support and dorsal neurocranium are more sensitive to Hh-attenuation than the ventral jaw, a hypothesis that is supported by the suggestion that dorsal and ventral mandibular identities are controlled by specific sets of transcription factors and signaling molecules [79]. This has been validated by the existence of a number of zebrafish jaw mutants where the dorsal and ventral structures within the first two arches are affected to differing degrees (*chameleon, sucker, schmerle*) [80].

Finally, our findings that Hh-signaling is required during gastrulation (4-10 hpf) for proper ventral jaw development is similar to previous findings that this time period is required for proper neurocranium development [18]. Thus, patterning of all anterior craniofacial structures (both dorsal and ventral) is likely controlled by the same Hh-signaling event that originates in the ventral brain primordium during gastrulation, as hypothesized by Eberhart et al., 2006. However, unlike dorsal neurocranium precursors that continue to require Hh-signaling for subsequent chondrogenesis up to 2 dpf [23], we show that jaw precursors require a single Hh-signaling event during gastrulation for both patterning and chondrogenesis (see Figures 8 and 9). Importantly, this early Hh-signal also influences later gene expression in the oral ectoderm, such as *pitx2 *[18] and *shh *itself (data not shown), which are good candidate genes for signals required by jaw precursors for CNCC chondrogenesis. However, more work will be required in order to determine the specific Hh-dependent signals for the proper development of both the neurocranium and jaw in the zebrafish.

### Hh-signaling is required for patterning the jaw joint region in the mandibular and hyoid arch

Our studies here reveal multiple requirements for *disp1 *and Hh-signaling in the development of the jaw joint region. Larval cartilage preparations show absence of the jaw joints, both bilaterally in the anterior arches and within the midline of the mandibular arch. Additional morphological defects included the loss of the RAP extension of the Mc element in the mandibular arch. These phenotypes were reminiscent of *bapx1*-morphant larvae [65]. *bapx1 *is expressed in arch mesenchyme and prospective joint domains within the first and second arch and its absence results in a failure of the joints to become properly specified, potentially through influencing *gdf5 *expression which is required in mouse appendicular and axial joint formation [63,66,67]. The joint specifying marker *bapx1 *was either reduced (bilaterally) or completely absent (midline) in *con/disp1 *mesenchyme. These *bapx1 *gene disruptions were sufficient to reduce *gdf5 *expression to barely detectable amounts in the *con/disp1 *embryos. Collectively, this data provides a genetic mechanism for Hh-signaling in both the development of jaw joint regions as well as the ventral midline bh element, which is also lost in *con/disp1 *larvae and requires *gdf5 *function [65].

Furthermore, careful analysis of the bilateral joints in the hyoid arch revealed two consistent, varying phenotypes: either ectopic cartilage cells forming an aberrant joint between the hs and ch elements or the complete absence of cartilage cells at the would-be joint site, leading to the failure of hs and ch elements to properly articulate. The presence of these two phenotypes in the mutant hyoid arch cartilages likely reflects two separate requirements for Hh-signaling in CNCC and neighboring cells that reside within future joint sites; first, in CNCC chondrogenesis to promote cartilage formation (as described in Figures 1 and 3) and secondly in joint organization within cells that will eventually chondrify (as described above). The different mutant phenotypes may reflect very slight differences in the amount of Hh-signaling present in the joint regions, as we occasionally visualized both of the described PA2 mutant phenotypes occurring on opposite sides of the same *con/disp1 *larvae (left and right sides of single larvae shown in Figure 6J, K).

### Hh-signaling controls chondrogenic differentiation of CNCC in the posterior arches

Unlike *con/disp1 *CNCC that have migrated into the mandibular arch, CNCC migrating into the hyoid and posterior arches condense normally. These crest cells become segmentally patterned within well-formed endodermal pouches, indicating that Hh-signaling is not involved in endodermal pouch morphogenesis nor is it required for invading CNCC to adhere to the PA2-7 epithelia. However, our studies reveal that those cells residing in the posterior arches require Hh-signaling to undergo chondrogenic differentiation. This is not the case in the hyoid arch, however, wherein normal CNCC differentiation and subsequent chondrification occurs. Variations in the potential of PA2-7 CNCC to differentiate may be due to intrinsic differences in the ability of these cell groups to respond to Hh-signals, or Hh-dependent co-factor(s), in the facial primordia. CNCC-responsiveness to local facial patterning cues depends in part on their Hox gene expression, which is determined by the original location of these cells in the hindbrain. Moreover, Hox status differs between CNCC in the second arch (*hoxa2*) and the posterior arches (*hoxa2/hoxb3*) [81]. In chick embryos, grafts of facial ectoderm or foregut endoderm, into the PA2 region has no influence on the fate of this arch [4,21]. Rather, PA2 mesenchyme is thought to be influenced by the pharyngeal ectodermal margin, and *shh *from this tissue has been implicated (directly or indirectly) in hyoid outgrowth [82,83].

The absence of cartilage elements in the posterior arches of 96 hpf *con/disp1 *mutant larvae is not due to changes in cell survival or proliferation as we were unable to find any variation in cell death or cell proliferation in the craniofacial region between *con/disp1 *mutants and their wild type siblings. This is consistent with zebrafish studies utilizing both *smu/smo *mutants and Shha;Shhb double morphant embryos, which lack dorsal cartilages yet display no changes in cell death or proliferation [18,23]. Rather, we found that Hh-signaling is involved in maintaining the chondrogenic differentiation potential of posterior arch mesenchyme. Specifically, critical transcription factors, *sox9a *and *dlx2a*, become down-regulated in these cells during the second day of development. Notably, other critical transcription factors such as *hand2, barx1, msxb, msxe *were not downregulated in these cells indicating that these cells persist in the posterior arches in a differentiated state that still resembles their neural crest origin.

Genetic analysis on the *sox9a/jellyfish *(*jef*) mutant has revealed a critical function for this gene in cartilage development within the posterior arches, potentially by influencing normal *col2a1 *expression in a similar fashion to the *con/disp1 *mutant [13]. The critical importance of *sox9a *in directing PA3-7 prechondrogenic condensations to develop as chondrocytes is underscored by the findings that other chondrogenic factors, like *runx2b*, are expressed at normal levels in the *sox9a/jef *and *con/disp1 *mutants alike [[13] and our studies].

So what then happens to the mesenchyme within the posterior arches of *con/disp1 *mutants? Our histological sections on 5 dpf *con/disp1 *mutants suggest that CNCC in PA3-7 give rise to fibrous connective tissue, a common derivative of pluripotent mesenchymal neural crest cells. We hypothesize that these ectopic fibrous-connective tissue cells are of CNC origin and have committed to a mesenchymal lineage, instead of a neural one, independently of Hh-signaling. These cells then require the Hh-pathway to adopt, or maintain, a chondrogenic fate. Consistent with this, exogenous Shh can induce multipotent CNCC and pluripotent mesenchymal stem cells to adopt a chondrogenic fate *in vitro *[84-87]. Examining later skeletal development in the *con/disp1 *mutant pharyngeal arches is frustrated by early embryonic death by 6-7 dpf in these animals.

Our stage-specific Hh-signaling inhibition studies revealed that the critical time for Hh-signaling to maintain chondrogenic differentiation in posterior arch-residing CNCC is the late pharyngula stage (32-48 hpf). At this time, *shh *and *disp1 *are coexpressed in facial ectoderm and endoderm, as well as the ventral brain, making it difficult to interpret the crucial Hh-source for posterior arch cartilage formation. An intriguing tissue source of *shh *in this process is the pharyngeal endoderm. In *casanova *mutants, which fail to make endoderm, all PA-derived cartilages are lost [11]. Further, surgical removal of foregut endoderm in chick embryo prior to CNCC migration results in a loss of ventral arch structures, yet arch identity is restored when Shh-soaked heparin beads are placed in the region of the excised tissue [88]. More studies will be required to determine the cell autonomy of Hh-ligands in posterior arch cartilage formation, as well as whether the signal must be interpreted by mesenchymal-neural crest and/or the surrounding epithelia.

### Conserved and non-conserved roles for Hh-signaling in PA development

Zebrafish PA skeletal defects in *con/disp1 *mutants resemble loss-of-function mutations to Shh in mouse [89,90] and treatments with Shh-blocking-antibodies in chick embryos [7], indicating a conserved role for Hh-signaling in PA development. However, unlike in fish, the primary defect attributing to the loss of mouse and chick PA skeleton is a high degree of cell death in CNCC as they migrate into the pharyngeal pouches. Additionally in Shh^-/- ^mouse, further cell death is seen in the pharyngeal pouch endoderm [90], indicating a pro-survival role for Hh-signaling in epithelial and mesenchymal facial tissue.

Hh-signaling may mediate its pro-chondrogenic (zebrafish) or pro-survival (mouse, chick) effects within CNCC through its influence on *Sox9 *gene activity. We noticed a strong correlation between the loss of *sox9a *expression in *con/disp1 *CNCC and the failure of those same cells to chondrify. In Shh^-/- ^mouse there is a striking pattern of *Sox9 *gene expression loss in streaming CNCC just prior to cell death within the same regions [90]. Inverse experiments, where ectopic Shh was used to stimulate mouse or human chondrocytes revealed that increased levels of Shh and downstream target genes in chondrocytes correlated with higher levels of *Sox9 *expression [91,92]. In zebrafish, due to gene duplication there are two orthologs of Sox9, *sox9a *and *sox9b*, which are co-expressed in postmigratory-CNCC and they control chondrocyte morphogenesis and survival, respectively [13,44,93-95]. These roles are likely controlled by the single tetrapod *Sox9 *in higher vertebrates and can account for the cell death seen in these animals upon gene loss, whereas we show here that, for the most part, *sox9b *(survival) gene expression is independent of Hh-signaling in CNCC within the PA.

## Conclusion

While it has been previously established that Hh-signaling is required for craniofacial development, the characterization of the *con/disp1 *mutant has been invaluable for helping us determine precisely the requirements of Hh-signaling in ventral arch development. In full, we found that Hh-signaling is required for jaw size and outgrowth, jaw joint formation and for chondrogenesis within the posterior gill arches. The Hh-pathway controls CNCC patterning and differentiation in jaw precursors, but simply differentiation in gill cartilage precursors. We showed that multiple Hh-signaling events are required and these events are temporally separated and partly unique from those previously described for the neurocranium skeleton.

## List of abbreviations

AP: anterioposterior; bh: basihyal; cb: ceratobranchials; ch: ceratohyal; cl: cleithrum; CNCC: cranial neural crest cells; Cya: Cyclopamine; dpf: days post fertilization; Dispatched 1: Disp1; Hh: Hedgehog; HPE: Holoprosencephaly; hpf: hours post fertilization; hs: hyosymplectic; hy: hyomandibular element; m: prospective mouth/stomodeum; Mc: Meckel's cartilage; ML: mediolateral; ne: neuroectoderm; op: opercle; PA: pharyngeal arches; pq: palatoquadrate; ptp: ptyergoid process; oe: oral ectoderm; pe: pharyngeal endoderm; pem: posterior ectodermal margin; Shh: Sonic Hedgehog; Smo: Smoothened; sy: symplectic; tr: trabeculae.

## Authors' contributions

TS participated in the design of the study, carried out all the experiments and drafted the manuscript. SCA participated in its design and coordination and revised and approved the manuscript.

## Supplementary Material

Additional file 1 ***con/disp1*^*b*392 ^allele encodes a prematurely truncated *dispatched1***. (**A,B**) Ventral views of 4.5 dpf Alcian blue-stained cranial cartilage in *con/disp1*^*b*392 ^(A) and *con/disp1*^*tm*15*a *^(B) larvae are phenotypically similar. (**C**) Both *con/disp1 *mutant alleles reveal a similar decrease in whole-embryo gene transcript levels of Hh-signaling responsive gene *gli1 *by QPCR. In contrast, *disp1 *RNA levels are similar between *con/disp1 *mutants and their wild type siblings, indicating that *disp1 *gene levels are not sensitive to Hh-signaling in whole embryos. (**D**) Sequence traces of *disp1 *from the *con/disp1*^*b*392 ^allele reveal a premature stop codon in the sixth exon. (**E**) Schematic representation of the Disp1 protein. Arrows indicate sites of single base changes that introduce stop codons within *disp1 *gene as it corresponds to the mutant alleles used in this manuscript (*con/disp1*^*tm*15*a *^mutation described in [26]). (**F,G**) PCR genotyping strategy which utilizes a primer to introduce two unique nucleotides into Disp1 amino acids 262 and 263. These PCR generated mutations, when combined with the *con/disp1*^*b*392 ^C to T transition in amino acid 264, generates a unique *ClaI *recognition site. Contrary to this, the *ClaI *site is not created in a wild type allele (F). The *ClaI *recognition site co-segregates with the *con/disp1*^*b*392 ^mutant phenotype. Larvae were sorted at 48 hpf by characteristic mutant Shh-signaling axis defects, then genotyped for the *ClaI *RFLP by PCR and gel electrophoresis using primers within the genomic sequence of the sixth exon and subsequently digested by *ClaI*. *ClaI *digestion of the *con/disp1*^*b*392^mutant allele generates both 85 base pair (bp) and 30 bp fragments. *ClaI *fails to digest wild type alleles which results in an 115 bp fragment (G). The 30 bp fragments are not shown in (G) as they are hard to delineate within resulting primer dimers generated by the PCR and are unnecessary for our genotyping conclusions as the 85 bp fragment is sufficient. Click here for file

Additional file 2 **Specification and migration of CNCC occur normally in *con/disp1 *mutants**. Dorsal (A-D), or lateral (E,F) views of 13 hpf (A,B), 20 hpf (C,D), or 24 hpf (E,F) wild type (A,C,E) or *con/disp1 *mutants (B,D,F) embryos labeled with RNA probe to *foxd3 *(A,B), *crestin *(C,D) or *sox9a *(E,F). (**A-D**) The patterns are indistinguishable between wild type and *con/disp1 *mutants at 13 hpf (*foxd3*) and 20 hpf (*crestin*). (**E,F**) *sox9a *marks CNC condensations at 24 hpf in wild type embryos (E) and *con/disp1 *mutants alike (F). Click here for file

Additional file 3 ***con/disp1 *mutants do not display an increase in cell death within PA**. (**A,B**) Lateral views displaying TUNEL positive cells in 65 hpf wild type (A) or *con/disp1 *(B) embryos. Red arrows in (A,B) are showing a portion of the cells we counted positive to reflect a dying cell in the pharyngeal region. The pharyngeal region was defined by us to include all tissue ventral to the eye and otic vesicle and dorsal to the heart and yolk. (**C**) Graph displaying the mean number of dying cells (11.6) for 11 wild type embryos (standard deviation was 4.8) and the mean number of dying cells (13.25) for 12 *con/disp1 *mutants (standard deviation was 4.8). This was not considered significant (p-value > 0.05, actual p-value was 0.432) by a two-tailed student t-test. These results reflect a lack of cell death at 65 hpf, which is consistent with what we saw at earlier and later stages (not shown in this figure). Click here for file

Additional file 4 ***sox9b *and *barx1 *expression following early and late Cya treatments**. Lateral views of 60 hpf (A-C) or ventral views of 72 hpf (D-F) embryos that were untreated (A,D) or treated with Cya from 4-10 hpf (B,E) or 32-48 hpf (C,F) that are labeled with RNA probe for *sox9b *(A-C) or *barx1 *(D-F). (**A-F**) Cya treatments at these stages did not disrupt *sox9b *or *barx1 *expression patterns within mesenchymal condensation in PA. Click here for file
