# Supplementary figures and images for: Zebrafish con/disp1 reveals multiple spatiotemporal requirements for Hedgehog-signaling in craniofacial development
Source: BMC Dev Biol. 2009 Nov 30;9:59. doi: 10.1186/1471-213X-9-59 (PMC2791760; doi:10.1186/1471-213X-9-59)

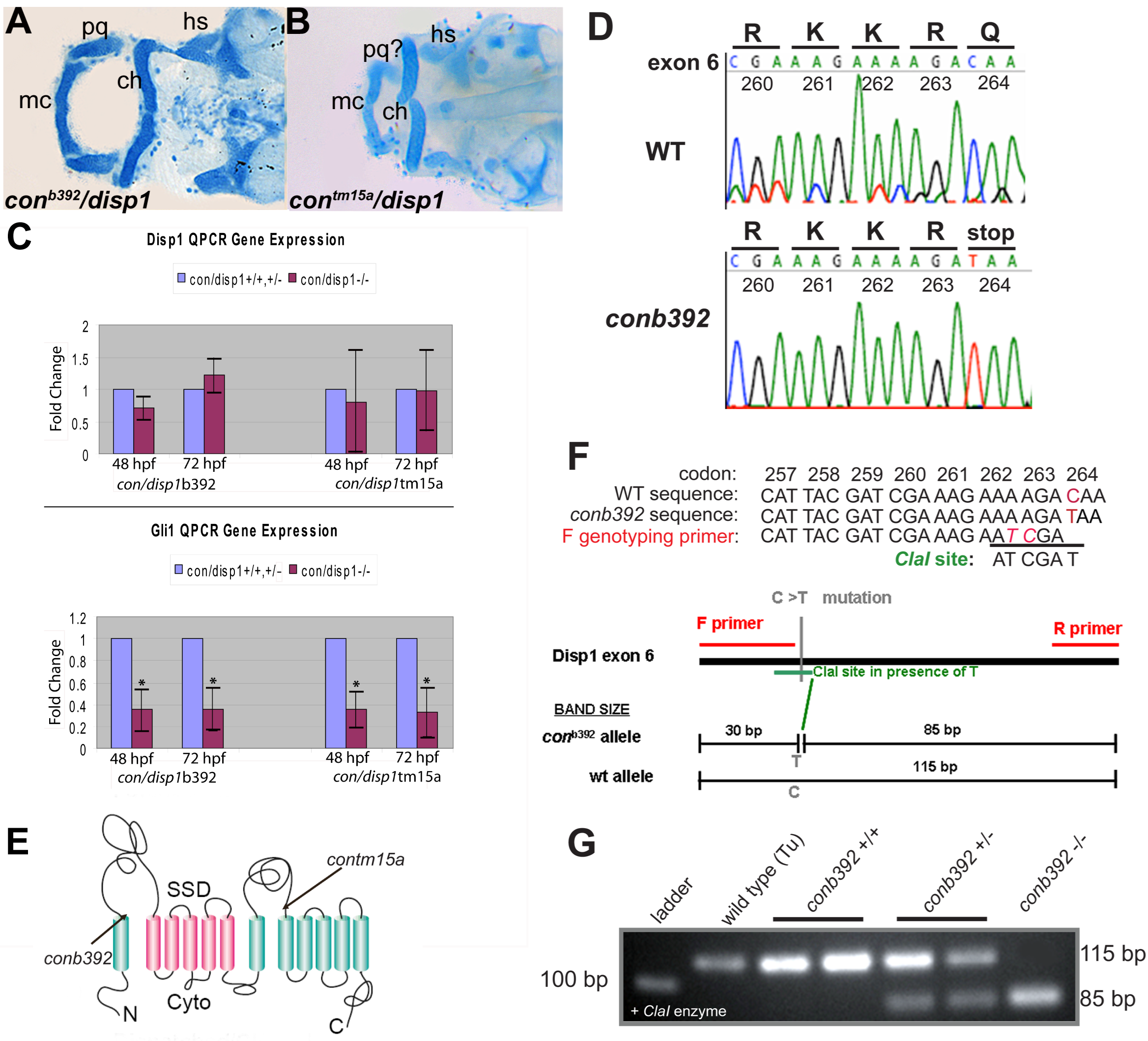

Supplement: Additional file 1 — con/disp1b392 allele encodes a prematurely truncated dispatched1. (A,B) Ventral views of 4.5 dpf Alcian blue-stained cranial cartilage in con/disp1b392 (A) and con/disp1tm15a (B) larvae are phenotypically similar. (C) Both con/disp1 mutant alleles reveal a similar decrease in whole-embryo gene transcript levels of Hh-signaling responsive gene gli1 by QPCR. In contrast, disp1 RNA levels are similar between con/disp1 mutants and their wild type siblings, indicating that disp1 gene levels are not sensitive to Hh-signaling in whole embryos. (D) Sequence traces of disp1 from the con/disp1b392 allele reveal a premature stop codon in the sixth exon. (E) Schematic representation of the Disp1 protein. Arrows indicate sites of single base changes that introduce stop codons within disp1 gene as it corresponds to the mutant alleles used in this manuscript (con/disp1tm15a mutation described in [26]). (F,G) PCR genotyping strategy which utilizes a primer to introduce two unique nucleotides into Disp1 amino acids 262 and 263. These PCR generated mutations, when combined with the con/disp1b392 C to T transition in amino acid 264, generates a unique ClaI recognition site. Contrary to this, the ClaI site is not created in a wild type allele (F). The ClaI recognition site co-segregates with the con/disp1b392 mutant phenotype. Larvae were sorted at 48 hpf by characteristic mutant Shh-signaling axis defects, then genotyped for the ClaI RFLP by PCR and gel electrophoresis using primers within the genomic sequence of the sixth exon and subsequently digested by ClaI. ClaI digestion of the con/disp1b392mutant allele generates both 85 base pair (bp) and 30 bp fragments. ClaI fails to digest wild type alleles which results in an 115 bp fragment (G). The 30 bp fragments are not shown in (G) as they are hard to delineate within resulting primer dimers generated by the PCR and are unnecessary for our genotyping conclusions as the 85 bp fragment is sufficient. [file 1471-213X-9-59-S1.TIFF]

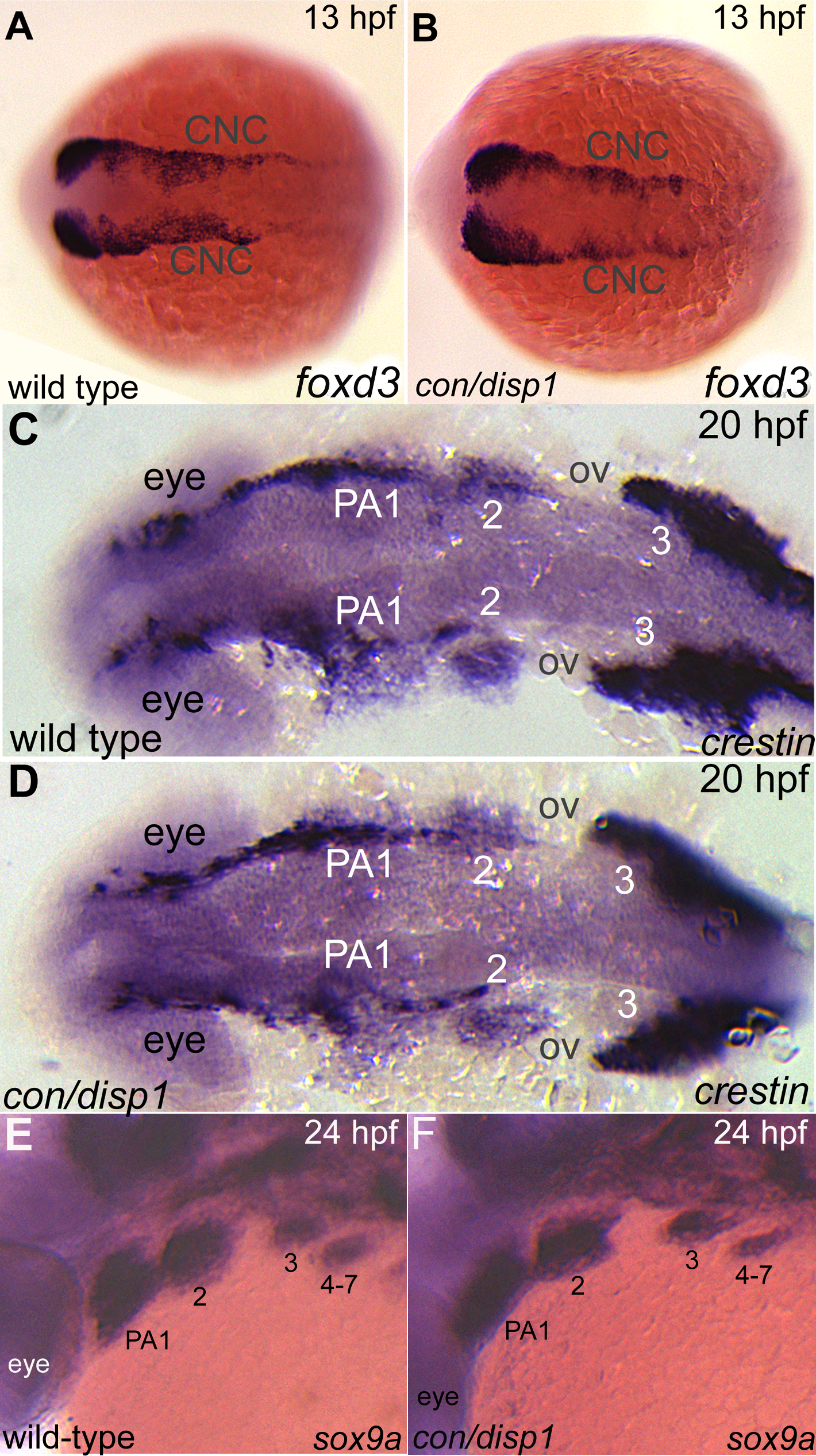

Supplement: Additional file 2 — Specification and migration of CNCC occur normally in con/disp1 mutants. Dorsal (A-D), or lateral (E,F) views of 13 hpf (A,B), 20 hpf (C,D), or 24 hpf (E,F) wild type (A,C,E) or con/disp1 mutants (B,D,F) embryos labeled with RNA probe to foxd3 (A,B), crestin (C,D) or sox9a (E,F). (A-D) The patterns are indistinguishable between wild type and con/disp1 mutants at 13 hpf (foxd3) and 20 hpf (crestin). (E,F) sox9a marks CNC condensations at 24 hpf in wild type embryos (E) and con/disp1 mutants alike (F). [file 1471-213X-9-59-S2.TIFF]

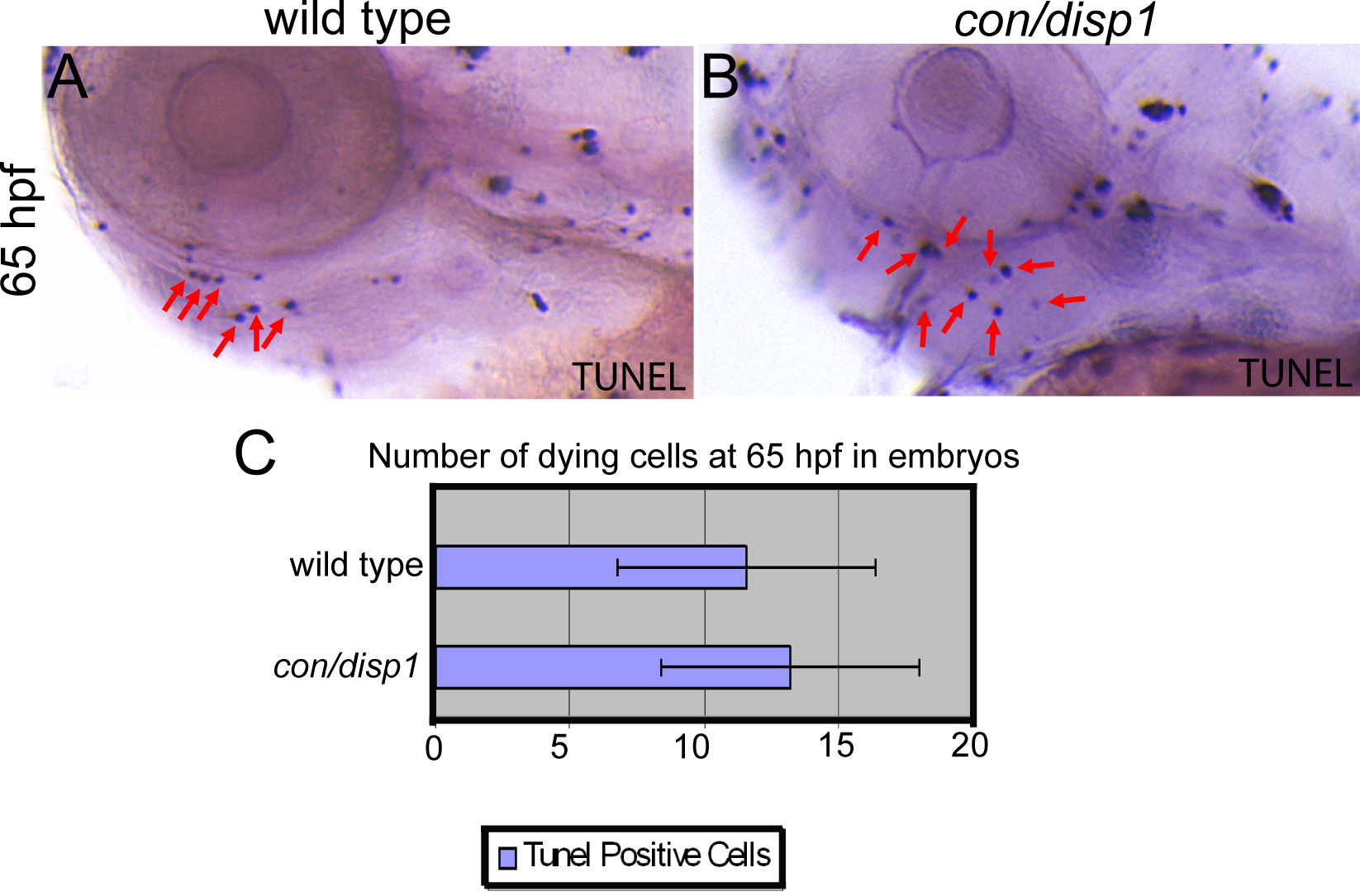

Supplement: Additional file 3 — con/disp1 mutants do not display an increase in cell death within PA. (A,B) Lateral views displaying TUNEL positive cells in 65 hpf wild type (A) or con/disp1 (B) embryos. Red arrows in (A,B) are showing a portion of the cells we counted positive to reflect a dying cell in the pharyngeal region. The pharyngeal region was defined by us to include all tissue ventral to the eye and otic vesicle and dorsal to the heart and yolk. (C) Graph displaying the mean number of dying cells (11.6) for 11 wild type embryos (standard deviation was 4.8) and the mean number of dying cells (13.25) for 12 con/disp1 mutants (standard deviation was 4.8). This was not considered significant (p-value > 0.05, actual p-value was 0.432) by a two-tailed student t-test. These results reflect a lack of cell death at 65 hpf, which is consistent with what we saw at earlier and later stages (not shown in this figure). [file 1471-213X-9-59-S3.TIFF]

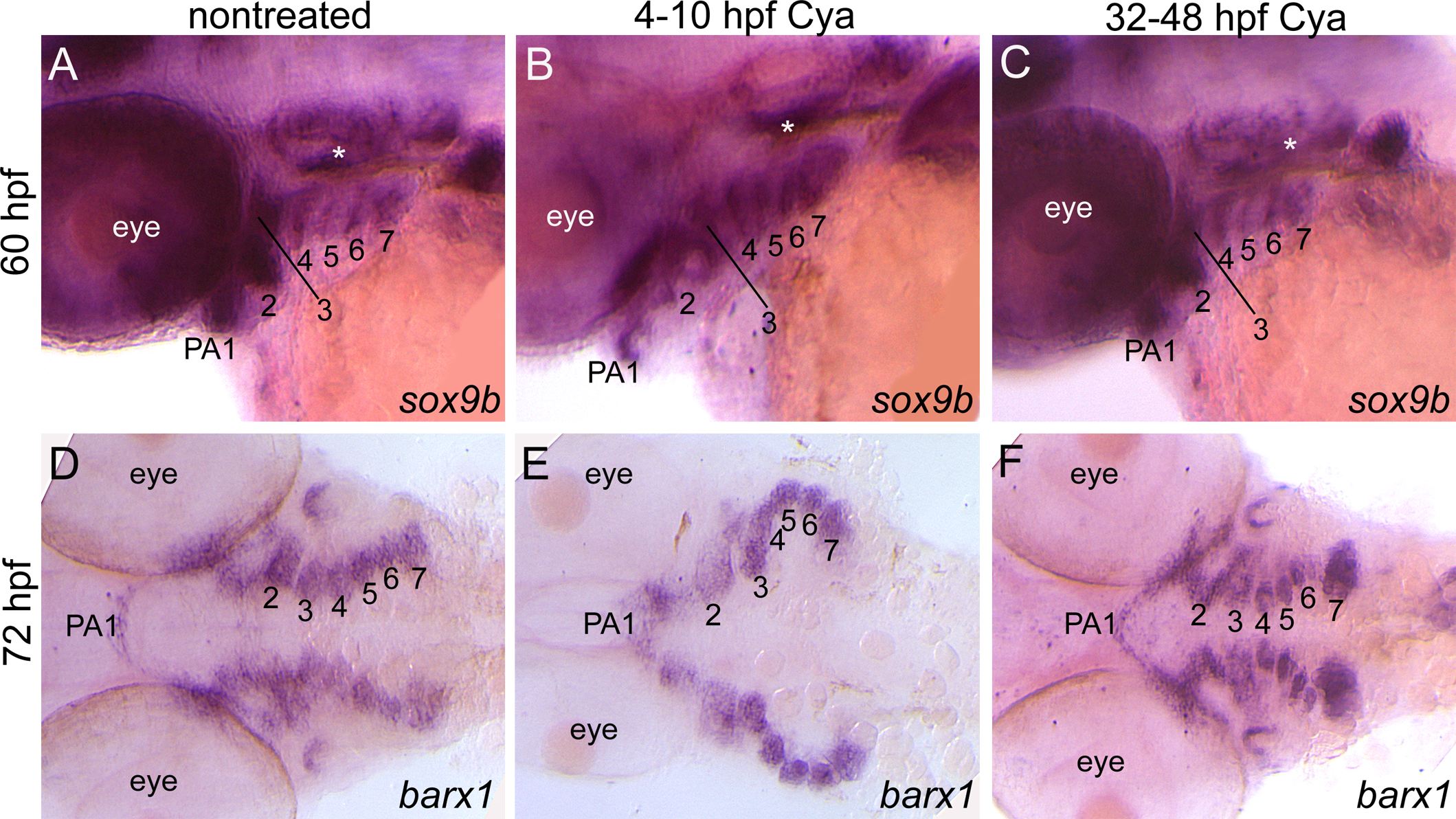

Supplement: Additional file 4 — sox9b and barx1 expression following early and late Cya treatments. Lateral views of 60 hpf (A-C) or ventral views of 72 hpf (D-F) embryos that were untreated (A,D) or treated with Cya from 4-10 hpf (B,E) or 32-48 hpf (C,F) that are labeled with RNA probe for sox9b (A-C) or barx1 (D-F). (A-F) Cya treatments at these stages did not disrupt sox9b or barx1 expression patterns within mesenchymal condensation in PA. [file 1471-213X-9-59-S4.TIFF]
